# Multivariate analysis and ADME profiling of *Rosmarinus officinalis* L. leaf vs. stem extracts: Extraction method-driven bioactivity and pharmacokinetic implications

**DOI:** 10.1016/j.bbrep.2026.102589

**Published:** 2026-04-16

**Authors:** Fatima-Zahrae Ed-darraz, Saida Tayibi, Sana Mounaimi, Asmae Hbika, Meryem Boufetacha, Karim Lyamlouli, Abdellatif Barakat, El Khadir Gharibi

**Affiliations:** a Laboratory of Applied Chemistry and Environment, Faculty of Sciences, Mohammed First University (UMP), Oujda, 60000, Morocco; b Agrobiosciences, College of Agriculture and Environmental Sciences (CAES), Mohammed VI Polytechnic University (UM6P), Ben Guerir, 43150, Morocco; c IATE, University of Montpellier, INRAE, Agro Institut of Montpelier, Montpellier, France

**Keywords:** Rosemary, Extraction, Pharmacokinetic, Antioxidant, Antimicrobial, Multivariate analysis

## Abstract

This study evaluated the bioactive potential of *Rosmarinus officinalis* L. extracts obtained separately from leaves and stems, comparing maceration (ME), Soxhlet (SE), and ultrasound-assisted extraction (UAE) techniques. Phytochemical profiling was performed using Liquid Chromatography-Electrospray Ionization-Tandem Mass Spectrometry (LC-ESI-MS/MS). Analyses quantified total phenols (TPC), flavonoids (TFC), antioxidant capacity (DPPH, FRAP, ABTS assays), and antimicrobial activity against *Escherichia coli* and *Streptomyces scabies*. Significant differences were observed between plant parts. SE of stems yielded the highest TPC (159.7 mg gallic acid equivalents/g). UAE most effectively recovered antioxidant diterpenes (e.g., carnosic acid, carnosol), producing the strongest antioxidant activity (DPPH IC_50_ = 11.95-16.30 μg/mL). Notably, stem extracts exhibited superior antimicrobial activity compared to leaf extracts, with UAE extracts demonstrating the highest efficacy against *S. scabies* (35 mm inhibition zone at 100 mg/mL). In silico absorption, distribution, metabolism, and excretion (ADME) predictions linked the lipophilicity (WLogP 3.25-5.87) of these diterpenes to their antimicrobial potency. Multivariate analyses clustered extracts primarily by extraction method and secondarily by plant part. Overall, fractionating rosemary into stems and leaves is crucial, as stems provide enhanced antimicrobial activity. UAE of stems is optimal for topical applications, whereas SE and ME may be more suitable for oral delivery.

## Highlights

• UAE optimizes diterpenes with superior antioxidant and antimicrobial effects.
• Soxhlet yields most phenolics but risks degradation of thermolabile compounds.
• Maceration enriches glycosides, suitable for oral delivery formulations.
• ADME links diterpenes to topical use and glycosides to encapsulated products.
• Multivariate analysis separates extracts by method and plant tissue type.

## Graphical abstract

**Figure:**
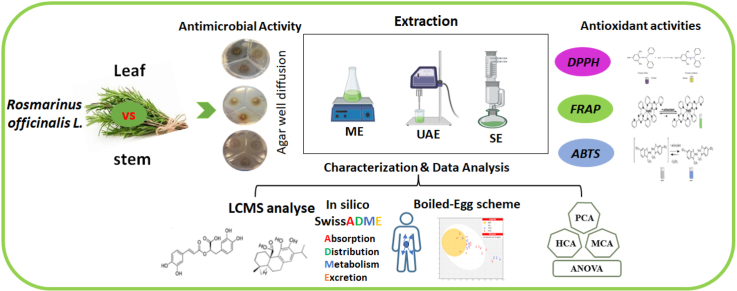

## 1 Introduction

*Rosmarinus officinalis* L. (syn. *Salvia rosmarinus Spenn*.), commonly known as rosemary, is a perennial aromatic shrub in the Lamiaceae family, recently reclassified into the genus *Salvia* thought phylogenetic analyses [1,2]. Native to the Mediterranean basin, resomary is characterized by upright stems (up to 1 m high), linear dark-green leaves, and whitish-blue inflorescences [3]. Historically, decoctions of its foliage have been used in folk medicine to treat parasitic infections, rheumatic disorders, and renal pathologies. Modern phytochemical studies have identified rosemary as a rich source of bioactive metabolites, underpinning diverse pharmacological properties, including antioxidants, antimicrobial, anti-inflammatory, anticancer, and antidiabetic effects [[4], [5], [6]]. Key phenolic compounds such as rosmarinic acid, carnosic acid, carnosol, and caffeic acid contribute to its potent antioxidant capacity [[7], [8], [9], [10]]. For example, Kumari Singh et al. [11] demonstrated that rosemary extract (1500 ppm) significantly improves oxidative stability in omega-3-rich structured lipids during accelerated storage, outperforming butylated hydroxytoluene (BHT) across multiple oxidation markers (PV, p-AV, TOTOX, CD, CT), highlighting its potential as a sustainable natural preservative.

The efficiency of bioactive compound recovery depends heavily on the extraction technique, which influences yield, composition, and bioactivity. Extraction methods determine the polarity range of extracted compounds, preserve thermolabile molecules, and the affect overall extraction kinetics [12,13]. Conventional and innovative techniques exhibit varying advantages depending on the chemical nature of bioactive constituents, matrix characteristics, and intended applications [14]. Regarding the extraction techniques, both conventional and innovative methods have been widely employed to recover antioxidants from rosemary. Soxhlet extraction (SE), a well-established traditional technique, demonstrates high efficiency in recovering compounds with moderate to low solubility [15]. In contrast, ultrasound-assisted extraction (UAE) utilizes high-frequency acoustic cavitation (20-40 kHz, 10-1000 W/cm^2^) to promote cell wall disruption through localized shear forces, microjetting, and thermal effects, thereby significantly enhancing solvent permeation [16]. While successive extraction by maceration (ME) remains widely applied due to its simplicity and low operational cost, its performance in terms of extraction yield, sustainability, and time efficiency is highly dependent on plant matrix characteristics and extraction conditions [17].

Although extensive research has focused on the phytochemical profile and bioactivity of rosemary leaves, stems-derived extracts, which are often discarded as agricultural by-products, remain largely underexplored in advanced phytochemical and biological studies. This study introduces an integrated and comparative approach by evaluating leaf and stem tissues through comprehensive phytochemical profiling combined with predictive ADME modeling, thereby addressing key gaps in the valorization of rosemary plant tissues. Specifically, this work aims to systematically assess the influence of plant part (leaves versus stems) and extraction technique (soxhlet extraction (SE), ultrasound-assisted extraction (UAE), and maceration extraction (ME)) on the chemical composition, antioxidant potential, and antimicrobial efficacy of hydroalcoholic rosemary extracts. Phytochemical characterization was performed using Fourier-transform infrared spectroscopy (FTIR) and liquid chromatography-mass spectrometry (LC-MS), and was further complemented by (silico) pharmacokinetic profiling of identified compounds via SwissADME software. Antioxidant potential was evaluated using multiple assays including ABTS + radical scavenging, ferric-reducing antioxidant power (FRAP), total phenolic content (TPC), and total flavonoid content (TFC), while antimicrobial activity was assessed against selected bacterial and fungal strains. The novelty of this study lies in its integrated and comparative evaluation of rosemary leaves and stems, employing three distinct extraction techniques and assessing both antioxidant and antimicrobial activities, strengthened by the combined application of ADME prediction, ANOVA, and multivariate statistical analyses to systematically evaluate biomass fractionation and its role in enhancing bioactive compound recovery.

## 2 Materials and methods

### 2.1 Plant material

*Rosmarinus officinalis* L. specimens were collected in Oulad M'hammed rural district ((Taourirt province, eastern Morocco; geographic coordinates: 33° 43′ 14″ North, 3° 01′ 36″ west, Average altitude: 1295 m) (see Fig. S1a in supplementary data). A voucher specimen of *Rosmarinus officinalis* L. was deposited at the Faculty of Sciences (Oujda) under accession code HUMPOM84 [18]. To preserve phytochemical integrity, leaves and stems were air-dried in the dark at ambient temperature (25 °C) to prevent photo-oxidative degradation. The dried biomass was homogenized using an electric grinder, sieved to a particle size <0.2 mm, and stored in airtight containers. Total solids content was quantified gravimetrically by oven-drying triplicate samples at 105 °C until constant weight (24 h), following ISO protocols [19]. Crude protein content was determined using the *Kjeldahl* method by calculating total nitrogen (N) and applying a conversion factor of 6.25 in accordance with ISO guidelines [20,21].

### 2.2 Extraction method

A comparative analysis of three extraction techniques (maceration (ME), ultrasound-assisted extraction (UAE) and Soxhlet extraction (SE)) was counducted to assess their effectiveness in recovering bioactive compounds from *Rosmarinus officinalis* L. (Fig. S1b). All methods employed a hydroalcoholic solvent system (50:50 V/V ethanol/water) to optimize polarity balance and extraction yield. For ME, 20 g of dried rosemary powder was mixed with 200 mL of solvent, and the mixture was magnetically stirred (600 rpm) for 24h. ME was performed at ambient temperature (25 ± 1 °C), whereas SE was carried out under reflux at 80 °C.

SE was conducted using a continuous reflux system with 20 g of dried powdered biomass loaded into a cellulose extraction thimble. The process employed 200 mL of solvent over a 6-h cyclic distillation without solvent renewal. UAE was performed using a suspension of 20 g of powdered biomass in 100 mL of solvent, with extraction carried out for 20 min using a 20 kHz, 500 W ultrasonic probe. The system operated in pulsed mode (20 s sonication, 10 s rest) at 60% amplitude, corresponding to an average power delivery of 200 W and a calculated power density of 2.0 W/mL within the solvent. A thermostated ice bath maintained the extraction temperature below 35 °C preventing solvent volatilization and thermal degradation of thermolabile compounds. Following extraction, mixtures were vacuum-filtered through Whatman No. 1 filter paper (11 μm pore size). All procedures were performed in three independent biological replicates (n = 3). Residual solids were oven-dried (50 °C, 24 h) to determine the extraction yields. Solvent removal from the combined filtrates was achieved by rotary evaporation (45 °C, 200 mbar), yielding concentrated extracts for subsequent analysis.

### 2.3 LC/ESI–MS/MS analysis of extract

Phytochemical profiling of rosemary leaf and stem extracts was performed using liquid chromatography/electrospray ionization–tandem mass spectrometry (LC/ESI–MS/MS). Analyses were conducted on a Shimadzu LCMS-8050 system (Shimadzu Corporation, Japan) equipped with a Zorbax Eclipse XDB-C18 analytical column (4.6 × 150 mm, 3.5 μm particle size; Agilent Technologies, USA). The system was operated using LabSolutions software (v.5.97), with a binary mobile phase gradient and optimized mass spectrometry parameters, as described in previous methodologies [22]. Ionization was carried out in negative polarity mode, with full scan mass spectra acquired over an *m*/*z* range from 100 to 1500. Data acquisition and spectral interpretation followed established protocols for phenolic compound identification.

### 2.4 Infrared spectroscopy characterization

Fourier transform infrared spectroscopy (FT-IR) analysis was performed using a JASCO FT/IR-470 spectrometer in the 4000 to 400 cm^−1^ range. Initially, an interferogram, corresponding to the detector signal as a function of time was recorded. The absorption spectrum was subsequently obtained by Fourier transforming of the interferogram.

### 2.5 Total phenolic and flavonoid content

#### 2.5.1 Total phenolic content (TPC)

The total phenolic content (TPC) was quantified using the Folin-Ciocalteu method [23]. Briefly, 20 μL of extract was mixed with 100 μL of 10% (v/v) Folin-Ciocalteu reagent and 80 μL of 7.5% sodium carbonate (Na_2_CO_3_) in the microplate. After 30 min incubation in darkness, absorbance was measured at 735 nm using a microplate reader (96 well culture plates), with gallic acid (0-250 μg/mL) used as the calibration standard.

#### 2.5.2 Total flavonoid content (TFC)

The total flavonoid content (TFC) was determined using the aluminum chloride colorimetric method (AlCl_3_) [24]. Briefly, 100 μL of extract was mixed with 50 μL of 1.2% (w/v) aluminum chloride hexahydrate (AlCl_3_·6H_2_O) and 50 μL of 120 mM potassium acetate (CH_3_CO_2_K) in microplate. The reaction mixture was incubated for 30 min at 23 ± 1 °C. Absorbance at 415 nm was measured, and TFC was expressed as quercetin equivalents (mg QE/g extract) using a standard curve (R^2^ = 0.998).

### 2.6 Antioxidant activity assays

Phenolic compounds exert their antioxidant activity primarily through two synergistic mechanisms: (1) hydrogen atom transfer (HAT) from hydroxyl groups to neutralize reactive oxygen species (ROS), and (2) single electron transfer (SET) process that interupt free-radical chain reactions [25]. These mechanisms are critical in food preservation, where oxidative degradation is inhibited, and in human health, where bioactive functionalities are promoted [26]. In this study, antioxidant capacity of *Rosmarinus officinalis* leaf and stem extracts was evaluated using DPPH, ABTS, and FRAP assays.

Natural antioxidants such as rosemary extracts significantly enhance the oxidative stability of emulsified oils, exhibiting antioxidant performance comparable to synthetic additives such as Butylated hydroxytoluene (BHT). Previous studies demonstrated that rosemary extracts markedly reduce peroxide values (PV) in sunflower oil, indicating lower oxidation rates [27]. For example, sunflower oil supplemented with 200 mg/kg of rosemary extract exhibited a PV of 75.7 meq/kg after three weeks, significantly lower than untreated controls. Rosemary extracts have also been shown to reduce oxidation markers such as TBARS and free fatty acids, performing similarly to or better than synthetic antioxidants and tocopherols [[27], [28], [29]]. In emulsified food systems, rosemary extracts improve oxidative stability during extended storage, maintaining product quality in items such as sausages [30]. Appropriate concentrations can therefore extend shelf life, offering a natural alternative to synthetic [29]. Nevertheless, antioxidant efficacy is concentration dependent and matrix-specific, highlighting the need for further optimization. Cited literature encompasses studies addressing these applications in real food systems.

#### 2.6.1 DPPH radical scavenging assay

Free radical scavenging activity (RSA) was evaluated against 2,2-diphenyl-1-picrylhydrazyl (DPPH) following a modified protocol [31]. Briefly, 100 μL of methanolic extract (100 μg/mL) was mixed with 100 μL of DPPH solution (4 mg/100 mL in methanol) in a 96-well microplate. After 30 min of incubation in darkness, absorbance was measured at 517 nm using a BMG LABTECH microplate reader. Radical scavenging activity (RSA) (%) was calculated using Equation (1):

(Eq.1) RSA(%)=(A(control)‐A(sample))/A(control)×100

Where, A_control_ and A_sample_ represent absorbance values of the DPPH solution without and with extract, respectively. All experiments were performed in triplicate [32].

#### 2.6.2 Ferric-reducing antioxidant power (FRAP)

The FRAP assay was conducted according to Kulichová et al. [33] the reaction mixture contained 30 μL of extract, 45 μL of 0.2 M HCl, 45 μL of 1% potassium ferricyanide, and 15 μL of 1% sodium dodecyl sulfate (SDS). After vortexing, 100 μL of distilled water (H_2_O) and 15 μL of 0.2% FeCl_3_ where added. Following 20 min incubation at 25 °C, absorbance was measured at 700 nm. Results were expressed as quercetin equivalents (mg QE/g extract) using a standard curve (0-100 μg/mL).

#### 2.6.3 ABTS ^+^ radical scavenging assay

ABTS^+^ [2,2′-azino-bis (3-ethylb enzothiazoline-6-sulfonic acid)] radical cation scavenging activity was assessed following Jo et al., [34] with modifications. ABTS solution (7 mM) was oxidized with 2.45 mM potassium persulfate for 16 h in darkness, then diluted to an absorbance of 0.70 ± 0.02 at 732 nm. Samples (20 μL) were mixed with 180 μL of diluted ABTS^+^, and absorbance was measured after 1 min. Results were expressed as ascorbic acid equivalents (mg AAE/g extract).

### 2.7 Antimicrobial activity evaluation

It should be noted that antimicrobial evaluation based solely on the agar well diffusion method presents inherent limitations. The absence of Minimum Inhibitory Concentration (MIC) determinations and a positive antibiotic control restrict the quantitative interpretation of antimicrobial potency.

#### 2.7.1 Microbial strains and culture conditions

The *in vitro* antimicrobial activity, expressed as zone diameter, is particularly relevant for topical applications. Antibacterial activity was tested against *Streptomyces scabies* (Luria Broth (LB); at 23 °C) and *Escherichia coli* (Mueller-Hinton (MHA) agar; at 37 °C). Culture Media were prepared according to Macwilliams and Liao [35] and Faye et al. [36], respectively.

#### 2.7.2 Agar-well diffusion assay

The agar-well diffusion assay [37] was performed with modifications. Bacterial Inocula were prepared by suspending colonies in 3 mL of 0.9% saline, followed by swabbing onto agar plates. Wells (8 mm diameter) were filled with 100 μL of extract (12.5-100 mg/mL in 5% DMSO). Plates were incubated for 24 h, and zones of inhibition (ZOI) were measured using a caliper. DMSO (5%) served as a negative control. Results are reported as mean ZOI ± SD from triplicate experiments.

Extracts were considered active when the inhibition zone diameter was greater than or equal to 15 mm [38].

### 2.8 ADME analysis

The pharmacokinetic profile of bioactive compounds, encompassing Absorption, Distribution, Metabolism, and Excretion (ADME), is critical determinant of therapeutic potential, governing bioavailability, tissue permeation, metabolic stability, and elimination kinetics. Both empirical and computational ADME assessment are integral to modern drug discovery, as early evaluation significantly reduces clinical attrition due to unfavorable pharmacokinetics [39,40]. Advances in computational modeling now allow high-throughput *in silico* ADME predicting, offering a cost-effective to experimental assays, particularly during early phytochemical screening. For plant-derived metabolites, such as those extracted from rosemary, *in silico* ADME modeling provides essential insights into bioavailability constraints, metabolic liabilities, and excretion pathways, thereby guiding structure-activity relationship optimization [41]. In this study, the SwissADME (http://www.swissadme.ch) was used to evaluate the pharmacokinetic behavior of the compounds identified in the extracts.

### 2.9 Data analysis

The average value were calculated for each treatment. Analysis of variance (ANOVA) was performed using R version 4.4.2. One-way ANOVA was used to assess the effect of extraction method on Total Polyphenol Content (TPC) and Total Flavonoid Content (TFC), while factorial ANOVA evaluated the combined effects of extraction technique and Plant-Part, followed by Tukey's HSD post-hoc test.

Multivariate analyses were conducted using XLSTAT (version 2016.02.28451), including Hierarchical Cluster Analysis (HCA), Principal Component Analysis (PCA), and Multiple Correspondence Analysis (MCA) were performed using OriginLab software to classify the six extraction modalities (LME, LUAE, LSE, SME, SUAE, SSE) into homogeneous groups based on the relative abundances of 32 identified metabolites.

## 3 Results and discussion

### 3.1 Proximate and spectroscopically analysis

Proximate analysis of *Rosmarinus officinalis* leaf and stem tissues revealed significant differences in moisture and crude protein content (Table 1). Stem samples exhibited lower moisture (6.78 ± 0.49%) and crude protein (5.21 ± 1.28%) than leaves (moisture: 7.97 ± 0.74%; crude protein: 7.40 ± 0.80%). Despite these differences, both parameters remained within a narrow biochemical range (<8%), suggesting organ-specific yet functionally compoarable chemical profiles [42]. Contrary to established mechanisms linking increased hydration to protein dilution [43], the observed data deviated from this inverse relationship. This discrepancy may reflect adaptive metabolic strategies in *Rosmarinus officinalis*, where resource allocation under xeric Mediterranean conditions favors the biosynthesis of stress-responsive secondary metabolites (e.g., terpenoids) over structural protein accumulation, as reported for arid-adapted perennials species [44].

**Table 1:** Yield and proximate composition of *Rosmarinus officinalis* L.

Parameters	Leaf	Stem
Moister content (%)	7.97 ± 0.74^a^	6.78 ± 0.49^b^
Protein calculated (%)	7.40 ± 0.80^a^	5.21 ± 1.28^b^
Yield (%)		
ME	20.1 ± 1.26^c^	14.1 ± 0.70^c^
UAE	15.7 ± 3.55^bc^	14.3 ± 2.95^c^
SE	11.9 ± 0.30^a^	7.24 ± 0.95^e^

ME: maceration extract; UAE: ultrasonic assisted extract; SE: soxhlet extract, Different superscript letters (^a^, ^b^) within a row indicate significant difference between Leaf and Stem (p < 0.05, *t*-test). For yield, different letters (^a^, ^b^, ^c^, ^d^, ^e^) across all six conditions indicate significant differences (p < 0.05, two-way ANOVA with Tukey's HSD).

Comparative evaluation of extraction yields demonstrated that leaf-derivied extracts consistently outperformed stem extracts across all extraction techniques (Table 1). For both tissues, yield followed the trend: Soxhlet extraction (SE) < ultrasound-assisted extraction (UAE) < maceration extraction (ME). Leaf yields (ME: 20.1 ± 1.26%; UAE: 15.7 ± 3.55%; SE: 11.9 ± 0.30%) exceeded stem yields, in agreement with previous studies (Balouiri et al. [4] and Vieitez et al. [45], who reported analogous results using methanol and 75% ethanol solvents, respectively. Maceration extraction (ME) produced the highest yields, however, literature reports demonstrate substantial methodological variability. Hosseini et al. [46] reported UAE ethanol yields of 20.8 ± 0.44% for leaves, whereas Genena et al. [47], observed higher SE yields (30.2%) using ethanol. Conversely, Zeroual et al. [24] reported superior SE performance (17.3 ± 1.09%) relative to ME (15.5 ± 0.81%) in cultivated rosemary. These discrepancies highlight the multifactorial nature of extraction efficiency, which is influenced by plant matrix composition (e.g. lignocellulosic structure, metabolite polarity), solvent affinity (polarity, dielectric constant), operational parameters (temperature, duration, solid-solvent ratio) and particle size [48,49].

ATR-FTIR spectra of rosemary leaves and stems (see Fig. S2 in supplementary data) reveal characteristic bands corresponding to the functional groups typical of lignocellulosic biomass [50]. The intensity of the absorption bands appearing in the leaf spectrum is comparable to that of the corresponding bands in the stem spectrum.

### 3.2 Total polyphenol (TPC) and flavonoid (TFC) content

**Fig. 1:**
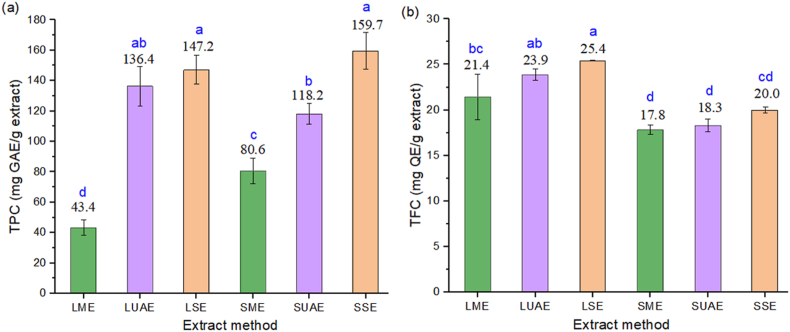
(a) Total phenolic content (TPC) and (b) total flavonoid content (TFC) in leaf and stem extracts. (GAE Gallic acid equivalent; QE Quercetin equivalent). The letters a,b, c, d indicate the statistical groupings using one-way ANOVA. LME: leaf maceration extract; LUAE: leaf ultrasonic assisted extract; LSE: leaf soxhlet extract; SME: stem maceration extract; SUAE: stem ultrasonic assisted extract; SSE: stem soxhlet extract.

*Rosmarinus officinalis* L. leaves exhibit high polyphenolic concentration attributable to their secondary metabolic profile [51]. Flavonoids, a class of phenolic compounds widely distributed in the plant kingdom, contribute to pigmentation in leaves, fruits, and flowers [52]. Ethanol was selected as the extraction solvent due to its intermediate polarity, which facilitates the solubilizing of hydrophilic phytochemicals [53]. Fig. 1 compares the total polyphenol content (TPC) and total flavonoid content (TFC) in leaf and stem extracts obtained via SE, ME and UAE extraction.

For TPC extraction, SE and ME yielded higher TPC values in stems compared to leaves, while UAE showed only marginal selectivity between plant tissues. In contrast, UAE preferentially extracted flavonoids from the stem, whereas SE and ME were more effective for the leaves.

Soxhlet extraction (SE) achieved significantly higher (p < 0.05) TPC values compared to other methods, with 136.38 ± 10.47 mg GAE/g in leaves and 159.7 ± 9.97 mg GAE/g in stems. For TFC, SE yielded maximum values in leaves (25.4 ± 0.04 mg QE/g), but stem flavonoids decreased to 20.0 ± 0.42 mg QE/g, suggesting possible thermal degradation at the 80 °C operating temperature. These results align with established literature demonstrating that, although elevated temperatures enhance polyphenol solubility [54], they simultaneously promote degradation of themolabile flavonoids [55]. The variation in extraction yield between plant tissues correlates with structural differences, particularly reduced diffusion efficiency in leaves associated with elevated cellulose content [56].

The global quantification of flavonoids, expressed as quercetin equivalents, reached its maximum in LSE, with the ranking is: SME(17.8) < SUAE(18.3) < SSE(20.0) < LME(21.4) < LUAE(23.9) < LSE(25.4). Meanwhile, global polyphenols quantification, expressed as Gallic acid equivalents, was highest in SSE (LME(43.4) < SME(80.6) < SUAE(118.2) < LUAE(136.4) < LSE(147.2) < SSE(159.7)). These findings highlight the significant influence of both extraction method and biomass fraction on TPC and TFC [57].

Analysis of variance (one-way ANOVA) revealed distinct statistical groupings in total phenolic content (TPC) across extraction methods and plants tissues. ANOVA was also applied to evaluate Extraction Method and Plant-Part Effects on yield and antioxidant assays, with results discussed below. Post-hoc testing (Tukey's HSD, p < 0.05) demonstrated that leaf Soxhlet extract (LSE) and stem Soxhlet extract (SSE) shared the same superscript letter (a), indicating no significant difference in their TPC means. Conversely, stem UAE (SUAE), stem maceration (SME), and leaf maceration (LME) exhibited distinct superscript letters (b, c, d), reflecting significant inter-group variance (p < 0.05). Leaf UAE (LUAE) displayed overlapping statistical significance (ab), indicating intermediate TPC values between SE and UAE derivied extracts.

For total flavonoid content (TFC), hierarchical clustering showed a different distribution pattern, with LSE and LUAE shared homogeneous subsets (a and ab), while SME and SUAE formed another overlapping group (d). LME and SSE exhibited significant pairwise differences (bc and cd), confirming method, and tissue, dependent variability in flavonoid recovery. These results underscore the critical influence of extraction methodology on phytochemical yield, with Soxhlet (SE) and ultrasound-assisted (UAE) techniques producing distinct metabolic extraction profiles.

### 3.3 Chemical composition of *Rosmarinus officinalis* L. extract

LC-MS analysis detected 71 compounds in rosemary extracts, with 32 metabolites identified (Table 2) and 39 remaining unidentified (Table S1 in supplementary data). The LC-MS spectra are presented in Fig. S3. Four diterpenes, carnosic acid, carnosol, methyl carnosate, and rosmanol, were consistently present across all extracts, regardless of extraction method or plant tissue origin. However, Table S1 shows that unidentified compounds were extract-specific, suggesting compositional variability. Numerous flavonoids and their glycosides were identified. The analysis revealed clear tissue-specific metabolite distribution. Leaf extracts contained unique metabolites including salvianolic acid B and isorhamnetin (identified in LUAE and LSE extracts), apigenin-7-*O*-glucoside (detected in LME and LSE extracts), and genkwanin (exclusive to LSE extracts). Stem extracts exhibited distinct compounds, particularly cirsimaritin (SME and SSE extracts), hesperidin-7-*O*-glucoside (SUAE and SSE extracts), and a unique phenanthrenone derivative (2,3,4,4a,10,10a-hexahydro-5,6-dihydroxy-1,1-dimethyl-7-(1-methylethyl)-9(1H)-phenanthrenone) detected exclusively in SME extracts.

**Table 2:** LC-MS identification of metabolites of the extract of rosemary leaf and stem.

	Compound	[M − H]^-^	*m*/*z* Main Fragments	LME	LUAE	LSE	SME	SUAE	SSE	Reference
		Class: Flavonoids								
Subclass: Flavone (Flavonoid)										
F1	Luteolin	285	175-133	0	X	X	X	0	0	[58]
F2	Apigenin	269	277-159-117	0	X	0	X	0	0	[59]
F3	Pectolinarigenin	313	283-255-163-117	0	0	X	0	X	0	[60]
F4	Cirsimaritin	313	283-255-163-117	0	0	0	X	X	0	[61]
F5	Genkwanin	283	268- 211- 172-117	0	0	X	0	0	0	[60]
Subclass: Flavone glycoside										
F6	Luteolin 7-*O*-rutinoside	593	503-473-383-353-279-194	X	X	X	X	X	X	[60]
F7	Luteolin-7-*O*-glucoside	447	285-199	0	X	0	0	X	X	[59]

F8	Apigenin-7-*O*-glucoside	431	430-369-269-253	X	0	X	0	0	0	[60]
Subclass: Flavanone glycoside										
F9	Hesperidin	609	301	0	0	X	X	X	X	[60]
F10	Homoplantaginin	462	463-419-329-285-135	X	0	0	0	0	0	[58]
Subclass: Flavone glucuronide										
F11	luteolin 3′-acetyl-*O*-glucuronide	503	285-255	0	X	0	X	X	X	[61]
F12	Luteolin 7-*O*-glucuronide	461	285-243-183-173	X	X	X	X	X	X	[60]
Subclass: Flavonoid										
F13	Gallocatechin	305	225-163-147	X	0	0	0	X	0	[62]
F14	Isorhamnetin	315	300-243-201-137	0	X	X	0	0	0	[60]
Subclass: Flavonoid glycoside										
F15	Hesperidin-7-*O*-glucoside	447	285-216-199-133	0	0	0	0	X	X	[60]
Subclass: Flavonol (Flavonoid)										
F16	Quercetin	301	285-241-202	0	0	0	X	X	X	[59]

**Class: Diterpenes**										

Subclass: Diterpene (Terpenoid)										
D1	Methyl carnostate	345	301-286-258	X	X	X	X	X	X	[62]
D2	Carnosic acid	331	287-227-199-134	X	X	X	X	X	X	[60]
D3	Carnosol	329	285-201	X	X	X	X	X	X	[58]
D4	Rosmanol	345	301-258	X	X	X	X	X	X	[58]
D5	Rosmadial	343	299-244-163-135	X	X	X	0	0	X	[58]
D6	Epirosmanol methyl ether	359	327-283-241-200	X	0	X	X	0	X	[62]
D7	Rosmanol quinone	343	299-244-163-135	X	X	X	0	0	0	[61]
D8	Rosmaridiphenol	315	285-257-183- 135	0	X	X	0	0	0	[61]
D9	2,3,4,4a,10,10a-Hexahidro-5,6-dihydroxy-1,1-dimethyl-7-(1-methylethyl)-9(1H)-Phenantrenone	301	258-189-123	0	0	0	X	0	0	[63]
D10	Epirosmanol	345	283-227	0	0	X	X	X	0	[62]
D11	Rosmanol methyl ether	360	361-315-231	0	0	X	0	0	X	[58]

**Class: Hydroxycinnamic acids**										

Subclass: Phenolic acid										
PA1	Caffeic acid	179	135	X	X	X	X	X	X	[59]
PA2	Rosmarinic acid	359	179-135	X	X	X	X	X	X	[58]
PA3	*p*-Coumaric acid	431	430-369	X	X	0	0	0	X	[64]
PA4	Salvianolic acid B	717	339-519	0	X	X	0	0	0	[60]
PA5	Neochlorogenic acid	353	191-135	0	0	0	X	X	X	[59]

X: Indicated the presence of the compound; F: Flavonoid; D: Diterpenes; PA: Phenolic acid; LME: leaf maceration extract; LUAE: leaf ultrasonic assisted extract; LSE: leaf soxhlet extract; SME: stem maceration extract; SUAE: stem ultrasonic assisted extract; SSE: stem soxhlet extract.

Extraction method significantly influenced compound detection. Maceration (LME) uniquely identified homoplantaginin, gallocatechin, and *p*-coumaric acid in leaf, whereas UAE revealed stem-specific compounds including pectolinarigenin, epirosmanol, and gallocatechin. Rosmanol quinone was consistently detected in all leaf extracts, while neochlorogenic acid and quercetin were present in all stem extracts regardless of the extraction method. These findings corroborate previous studies of rosemary phytochemistry [60,65].

Based on detected compounds, stem extracts demontrated greater metabolite diversity than leaf extracts. Among the extraction methods, Soxhlet extraction (SE) yielded the highest number of metabolites, following the ranking: LME (15) < SME (17) < LUAE (18) = SSE (18) = SUAE (18) < LSE (21). This method facilitated the extraction of high concentrations of key bioactive compounds, including rosmarinic acid, carnosic acid, carnosol, ursolic acid, and luteolin-7-*O*-glucoside acid [60].

Based on molecular class predominance, flavonoids were the dominant in UAE stem extracts, with the ranking: LME (5) < LUAE (7) = SSE (7) < LSE (8) = SME (8) < SUAE (10). Flavonoids are readily extractable from rosemary leaves, flowers, stem, and roots [66]. Diterpenes, particularly terpenoids, were most abundant in leaf extracts, with the ranking: SUAE (5) < SME (7) = LME (7) = LUAE (7) = SSE (7) < LSE (10). These compounds are less polar than glycosylated flavonoids, and are therefore efficiently extracted using the Soxhlet method. Ultrasound-assisted extraction can also be effective due to the cavitation phenomenon, whereas maceration is generally less efficient for extracting less polar coumpounds. Notably, the ranking of total flavonoid content differs from the overall flavonoid quantification expressed as quercetin equivalents which reached its maximum in the LSE. This discrepancy may be attributed to unidentified compounds (Table S1). Some of which may beling to flavonoid class.

Abietane diterpenoids, commonly found in *Rosmarinus officinalis* and characterized by a tricyclic structure [67], were identified in the extracts. Conversely, the phenolic acid subclass exhibited the lowest molecular abundance among the identified compounds. No significant distribution differences were observed for this subclass between plant tissue or extraction methods (LME (3) = LSE (3) = SME (3) = SUAE (3) < LUAE (4) = SSE (4)).

Among the compounds detected in all six extracts, diterpenes including methyl carnostate, Carnosic acid, rosmanol, and carnosol were predominant, followed by phenolic acids (caffeic acid and rosmarinic acid) and flavonoids (Luteolin 7-*O*-glucuronide and Luteolin 7-*O*-rutinoside). These bioactive compounds are consistently reported across rosemary extracts [61]. Among compounds identified in four extracts, two diterpenes (Rosmadial and Epirosmanol methyl ether) and two flavonoids (Hesperidin and luteolin 3′-acetyl-*O*-glucuronide) were preferentially extracted from the stems. Notably, luteolin 3′-acetyl-*O*-glucuronide was reported as the second most abundant compound in rosemary leaves collected from the Regenerative Organic Farm, Maharishi University of Management, Fairfield, Iowa, USA, during the year 2018 [68]. Epirosmanol methyl ether has also been identified in rosemary extracts from various Serbian provinces [63]. The ME, UAE and SE methods demonstrated similar extraction profiles for carnosic acid, carnosol, and related compounds. However, UAE extracts, characterized by elevated concentrations of lipophilic diterpenes such as carnosic acid, exhibited the highest antioxidant activity. This profile makes them particularly suitable for applications in lipid-based applications, such as natural preservatives in edible oils or topical formulations, supported by their efficacy in stabilizing omega-3 rich lipids [11]. ME extracts, enriched in soluble glycosides, are well-suited for encapsulated formulations designed to enhance bioavailability and enable oral administration. In contrast, LME exhibited an absence of poorly soluble flavonones. Overall, ME appears to favor the extraction of more polar compounds, such as glycosylated flavonoids and rosmarinic acid, while being less effective for less polar constituents like diterpenes. UAE, emerges as the most versatile method, enabling extraction of a broad spectrum of compounds, including flavonoid aglycones and glycosides, diterpenes, and phenolic acids due to the enhanced solvent penetration and compound release induced by cavitation. Soxhlet extraction (SE) is also effective for isolating diterpenes and flavonoids, although prolonged exposure to elevated temperatures may promote degradation of heat-sensitive compounds.

### 3.4 ATR-FTIR analysis

The ATR-FTIR spectra of rosemary leaves and stems extracts (Fig. S2) reveal characteristic bands corresponding to the functional groups of the compounds identified in LC-MS/MS (flavonoids, diterpenes, phenolic acids) (Table 2).

A broad and intense band centered around 3315.9 cm^−1^ is attributed to O–H stretching vibrations, which are characteristic of phenolic acids (e.g., rosmarinic acid, caffeic acid), flavonoids (e.g., luteolin, apigenin), and phenolic diterpenes (e.g., carnosol, rosmanol) [69,70]. The broad nature of this band may also result from residual moisiture, despite prior drying steps [71]. Sharper bands observed at 2932.2 cm^−1^ correspond to C–H stretching vibrations of alkyl groups, which are common in all organic compounds [72]. However, this region is generally less informative for differentiating between extracts due to the ubiquitous presence of aliphatic chains in plant derived matrices.

Bands around 1599.6 cm^−1^, and 1510.9 cm^−1^ are attributed to aromatic ring vibrations and C=C stretching vibrations of alkenes, confirming the presence of phenolic compounds [70].

A band at 1369.2 cm^−1^ corresponds C–H bending vibrations of alkanes. The band at 1260.2 cm^−1^ is characteristic of C–O stretching in esters, while the band at 1028.8 cm^−1^ is associated with C–H vibrations. Additionally, the band near 1026 cm^−1^ is attributed to deformation vibration of C–H bonds in aromatic rings, which are common constituents in rosemary [73]. A distinct band at 808.9 cm^−1^ indicates the presence of alkanes (=C–H). Overall, the observed O–H, C=C and C–O bands corroborate the presence of polyphenolic, aromatic and glycosylated structures, while aliphatic C–H absorptions reflect the presence of alkanes chains and methyl/methylene groups within diterpenes.

Table 3 summarizes the correspondence between the main FTIR absorption bands and their ranking according to intensity across the six extracts. Although FTIR analysis does not provide precise quantification determination of individual flavonoids, it offers valuable insight into functional groups characteristic of these compounds, including C=O stretching, C=C aromatic vibrations, and C–O stretching. The intensity ranking of the bands at 1599.6 cm^−1^, and 1510.9 cm^−1^ can be semi-quantitatively correlated with the total flavonoid concentration (TFC) (Table 2). when comparing rosemary plant tissues, leaves exhibit a higher concentration of flavonoids and phenolic acids, whereas stems are relatively enriched in specific diterpenes.

**Table 3:** Absorption band of rosemary leaves and stems.

Wave (cm^−1^)	Assignment	Reference	Class	Ranking order by band intensity
3315.9	O–H stretching bands (alcohol/phenols)	[69,70]	Flavonoids (luteolin, apigenin) Diterpenes (carnosol, rosmanol), Phenolic acids (rosmarinic acid, caffeic acid)	LSE(14.3) < SSE(19) < SME = LME(20) < LUAE(20.7) ≈ SUAE(21)
2932.2	C–H stretching (Alkanes)	[69,70]	Diterpenes (methyl carnosate, carnosic acid) Glycosides (luteolin 7-*O*-rutinoside). phenolic acids	LME(3.8) < SUAE(4.5) ≈ SSE(4.8) ≈ LUAE(5) < SME(7.8) < LSE(11)
1687.1	C=O and C=C stretches	[67]	All phenolic compounds (flavonoids, phenolic acids, phenolic diterpenes).	SUAE(2.5) ≈ SSE = LUAE (2.7) < LME (7.9) < SME(10.7) < LSE(12)
1599.6	C=C aromatic stretching	[70]	Flavonoids (e.g., luteolin, quercetin) Hydroxycinnamic acids (rosmarinic acid).	SME(17) ≈ LSE(17.2) < SUAE(19) < LUAE = SSE(20) < LME(23)
1510.9	C=C stretching	[70]	Flavonoids (luteolin, quercetin) Hydroxycinnamic acids (e.g., rosmarinic acid).	LSE(5.8) < SME = SUAE(7) < LME(8.2) < LUAE(8.8) ≈ SSE(9)
1369.2	O–H in-plane bending vibration	[67]	Hydroxycinnamic acids (rosmarinic acid).	SME(7) < SUAE(8) < SSE(11) < LSE(12.6) ≈ LUAE(12.7) < LME(14)
1260.2	C–O stretching (Esters/ethers)	[70]	glycosides (apigenin-7-*O*-glucoside) Flavonone (cirsimaritin and pectolinarigenin) diterpenes (epirosmanol methyl ether)	SUAE(9.8) < SSE(13) < SME(14) < LUAE(16.6) ≈ LSE(16.7) < LME18.5)
1028.8	C–H bending aromatic	[73]	flavonoids (luteolin) phenolic acids (caffeic acid)	LSE(23.4) < SSE=SUAE(29) < LME(30.8) < LUAE(31.2) < SME(34)
808.9	= CH out-of-plane bending (alkenes)	[74]	Diterpenes (carnosol)	LSE(3.5) ≈ SSE(3.6) < LUAE = SUAE(4) < LME (5.7) ≈ SME(6)

LME: leaf maceration extract; LUAE: leaf ultrasonic assisted extract; LSE: leaf soxhlet extract; SME: stem maceration extract; SUAE: stem ultrasonic assisted extract; SSE: stem soxhlet extract.

Regarding extraction methods, ME ranks highest for flavonoid extraction, as indicated by the strong bands at 1599.6 cm^−1^ (23) and 1510.9 cm^−1^ (8.2), as well for phenolic acids, reflected by bands at 1260.2 cm^−1^ (18.5) and 1369.2 cm^−1^ (14) in leaf extracts. SE, on the other hand, is particularly effective for oxygenated diterpenes such as carnosic acid, as evidence by the band at 1687.1 cm^−1^ (12) in leaf extracts. UAE demonstrate high efficiency in extracting hydroxylated compounds (flavonoids and phenolic acids), as shown by the high-intensity band at 3315.9 cm^−1^ (≈21), and for alkene-type diterpenes in stems, indicated by the band at 808.9 cm^−1^ (6).

### 3.5 Antioxidant activity of rosemary leaf and stem extracts

Table 4 presents the antioxidant activities of rosemary leaf and stem extracts, as assessed by DPPH, FRAP, and ABTS assays. The variations in antioxidant potential among the different extraction methods (ME, UAE, SE) can be partially explained by the different concentrations of the main antioxidant compounds extracted by each method.

**Table 4:** Antioxidant activity of rosemary extracts.

Method	DPPH (IC_50_, μg/mL)	FRAP (mg QE/g)	ABTS (mg AA/g)
**Leaf**			

ME	70.1 ± 0.03	45.4 ± 0.003	152.3 ± 0.002
UAE	16.3 ± 0.01	215.4 ± 0.06	179.2 ± 0.001
SE	9.92 ± 0.01	206.8 ± 0.22	179.5 ± 0.003

**Stem**			

ME	37.7 ± 0.02	126.3 ± 0.31	177.4 ± 0.001
UAE	11.9 ± 0.01	171.0 ± 0.08	178.1 ± 0.002
SE	33.7 ± 0.003	198.5 ± 0.02	179.2 ± 0.001

AA: Ascorbic acid, QE: Quercetin equivalent; LME: leaf maceration extract; LUAE: leaf ultrasonic assisted extract; LSE: leaf soxhlet extract; SME: stem maceration extract; SUAE: stem ultrasonic assisted extract; SSE: stem soxhlet extract.

In rosemary leaves, ME resulted in the highest IC_50_ value (70.1 ± 0.03 μg/mL), indicating the lowest antioxidant activity, despite a relatively high phenolic content [75]. In contrast, UAE and SE demonstrated significantly lower IC_50_ values (16.3 ± 0.01 μg/mL and 9.92 ± 0.01 μg/mL, respectively), indicating superior radical-neutralizing efficiency. These results reflect the principle that lower IC_50_ values correspond to greater antioxidant potency, as a smaller concentration is required to neutralize 50% of the free radicals [76]. These findings are consistent with those of Genena et al. [47], who reported an IC_50_ of 15.7 μg/mL for rosemary leaf extracted using SE. For stem extracts, a similar trend was observed. ME showed the highest IC_50_ value (37.7 ± 0.02 μg/mL), whereas UAE and SE exhibited more potent antioxidant activity, with IC_50_ values of 11.9 ± 0.01 μg/mL and 33.7 ± 0.003 μg/mL, respectively. Thus, UAE and SE methods outperformed ME, with UAE achieving the most efficient extraction for stems.

FRAP analysis revealed reducing powers ranging from 45.4 ± 0.003 mg QE/g in macerated (ME) leaf extracts to 206.8 ± 0.22 mg QE/g in SE leaf extracts. Stem extracts showed a similar pattern, varying from 126.3 ± 0.31 (ME) to 198.5 ± 0.02 mg QE/g (SE). The FRAP values obtained with UAE and SE leaf extracts were notably higher than those from ME extracts.

ABTS assays yielded radical-scavenging capacities ranging from152.3 ± 0.002 mg AA/g (ME leaf extract) to 179.5 ± 0.003 mg AA/g (UAE and SE leaf extracts) and 177.4 ± 0.001 (ME stem extract) to 179.2 ± 0.001 mg AA/g (SE stem extracts). The highest ABTS activity observed in SE leaf extract is consistent with their enrichment in methylated flavonoids (e.g., genkwanin) and oxidized diterpenes (e.g., rosmanol). Likewise, the combination of the lowest DPPH IC_50_ values and the high FRAP/ABTS values of UAE leaf extracts can be attributed to their elevated levels of diterpenes (carnosic acid, carnosol) and aglycone flavonoids (luteolin). In contrast, the moderate FRAP activity of SE stem extracts appears to be associated with the predominance of flavonoid glycosides (luteolin-7-*O*-rutinoside) and phenolic acids (rosmarinic acid).

The UAE and SE methods produced statistically comparable antioxidant activities across assays, while extracts derived from ME have the lowest antioxidant properties. The strong antioxidant activity of UAE and SE extracts is largely attributed to their higher diterpenes content. These findings corroborate prior studies emphasizing UAE's efficacy in releasing antioxidant phytochemicals, likely due to enhanced cell wall disruption and solvent penetration [4].

### *3.6* **Antimicrobial activity**

**Fig. 2:**
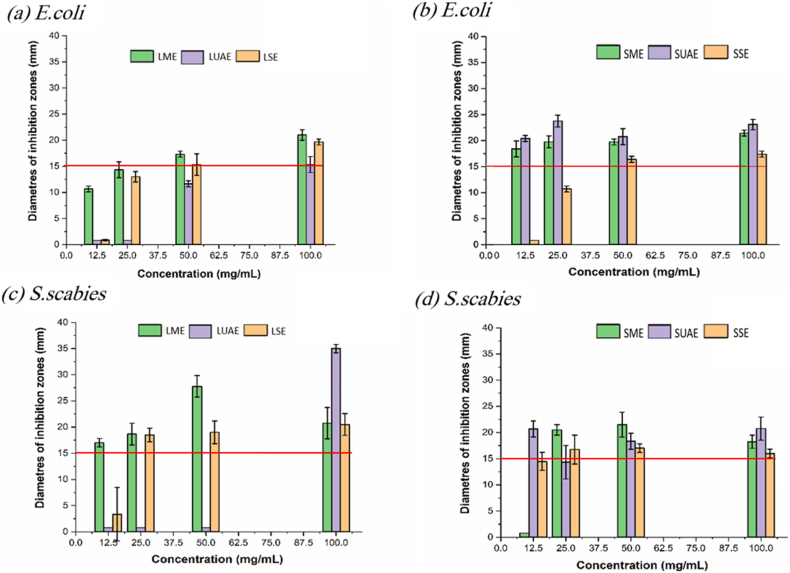
Diameters of inhibition zones (mm) for (a) (Leaf) and (b) (Stem) *Escherichia coli* and (c) (Leaf) and (d) (Stem) *Streptomyces scabies* bacteria. LME: leaf maceration extract; LUAE: leaf ultrasonic assisted extract; LSE: leaf soxhlet extract; SME: stem maceration extract; SUAE: stem ultrasonic assisted extract; SSE: stem soxhlet extract.

The *in vitro* antimicrobial activity of rosemary extracts, obtained through different extraction techniques, was evaluated using the agar well diffusion method against *Streptomyces scabies* and *Escherichia coli* (Fig. 2). The results varied depending on the extraction method and plant part.

Rosemary extracts showed antibacterial activity against both bacterial strains tested. The negative control (5% DMSO) exhibited no inhibitory effect. However, several methodological limitations constrain the interpretation of these results. The agar-well diffusion method employed serves as a qualitative screening assay only. Minimum inhibitory concentration (MIC) and minimum bactericidal concentration (MBC) values were not determined. No positive antibiotic control was included in the experimental design, and only two bacterial strains were evaluated. These constraints limit the robustness of the antimicrobial findings. The results should therefore be considered preliminary, and confirmation using standardized quantitative assays is required. Broth microdilution methods for MIC and MBC determination are recommended. Additionally, expanded strain panels representing diverse bacterial species should be evaluated. Such validation would establish the clinical and practical relevance of the observed antibacterial properties. As previously reported, rosemary extract macerated with 75% ethanol is a good source of antimicrobial compounds [45]. Fig. 2 shows that the inhibitory effect varies depending on the extraction method, bacterial species, and plant part (leaf or stem). In general, Stem extracts showed slightly stronger antimicrobial effects, particularly against *E. coli*. The UAE leaf extract displayed the highest inhibitory activity against *S. scabies* at 100 mg/mL.

As shown in Fig. 2a, ME leaf extracts demonstrated a gradual increase in *E. coli* inhibition starting at 25 mg/mL, suggesting a dose-dependent response. In contrast, SE extracts showed activity only above 50 mg/mL, whereas UAE extracts exhibited inhibitory effects only at 100 mg/mL.

For stem extracts (Fig. 2b), the antimicrobial activity appeared at lower concentration. ME and UAE extracts showed inhibition starting at 12.5 mg/mL. SE stem extracts displayed activity starting at 50 mg/mL, with a modest increase at higher concentrations. With regard to the inhibitory effects againts *S. scabies*, the ME leaf extracts (Fig. 2c) inhibited growth at 12.5 mg/mL, peaked at 50 mg/mL, and declined sharply at 100 mg/mL. The SE extract showed modest inhibition starting at 50 mg/mL and stabilized thereafter. Remarkably, the UAE extract only exhibited activity at 100 mg/mL, achieving the highest inhibition diameter (35 mm) across all tests. For the stem ME extracts (Fig. 2d), antimicrobial activity was delayed, emerging only at 50 mg/mL and stabilizing at higher concentrations. In contrast, both SE and UAE extracts maintained stable inhibition across the concentration range from 12.5 to 100 mg/mL, with UAE showing marginally greater efficacy. These findings are consistent with those of Moreno et al. [77], who evaluated the antibacterial activity of *Rosmarinus officinalis* extracts prepared using various organic solvents. Their results demonstrated that methanolic extracts exhibited significant inhibitory effects against *E. coli* using the disk diffusion method, outperforming extracts obtained with acetone or water. Rosemary leaf and stem extracts are known to be rich in secondary metabolites, such as phenolic compounds, including diterpenes, carnosol, carnosic acid, methyl carnosate, rosmanol, and epirosmanol, and phenolic acids such as ferulic, rosmarinic, chlorogenic and caffeic acids, which possess various biological activities, including antioxidant and antimicrobial properties [78]. Indeed, some of these polyphenols can inhibit bacterial growth by directly altering membrane fluidity, followed by physical rupture of the membrane, leakage of cytoplasm contents, and outflow of intracellular components [79]. Among the extraction methods tested, UAE yielded the highest antimicrobial activity for both leaf and stem extracts.

### 3.7 ADME modeling results

The observed antioxidant and antimicrobial activities of rosemary leaf and stem extracts can be attributed to the presence of various phenolic compounds and diterpenes identified via LC-ESI-MS/MS (Table 2). However, the *in vivo* efficacy of these bioactive compounds depends not only on their *in vitro* activity but also on their pharmacokinetic profiles, including absorption, distribution, metabolism, and excretion (ADME) [80,81]. Table 5 presents predicted ADME parameters for the major identified compounds, generated using SwissADME tool. While these predictions require experimental validation, they provide a useful preliminary framework for evaluating the correlation between bioactivity and potential bioavailability. ADME outputs are strictly in *silico* predictions and should be interpreted as testable hypotheses requiring experimental validation; likewise, multivariate analyses (PCA/HCA/MCA) are presented as exploratory, given the limited number of extracts (n = 6) relative to the metabolite space. All analyzed compounds exhibit an Esol (estimated solubility) greater than - 3.5, suggesting good water solubility. Moreover, compounds with higher Wlog P values exhibit high lipophilicity.

**Table 5:** Evaluation of the pharmacokinetic properties (ADME) of compounds in *Rosmarinic officinalis L*.

N	Compound	Score	Physicochemical Properties				Lipophilicity		
			Csp3	HBA	HBD	TPSA (Ả^2^)	XlogP3	WlogP	MlogP
Class: Flavonoids									
Subclass: Flavone (Flavonoid)									

F1	Luteolin	3	0	6	4	111,13	2,53	2,28	−0,03
F2	Apigenin	2	0	5	3	90,9	3,02	2,58	0,52
F3	Pectolinarigenin	2	0,12	6	2	89,13	3,32	2,89	0,47
F4	Cirsimaritin	2	0,12	6	2	89,13	3,32	2,89	0,47
F5	Genkwanin	1	0,06	5	2	79,9	3,35	2,88	0,77
Subclass: Flavone glycoside									
F6	Luteolin 7-*O*-rutinoside	6	0,44	15	9	249,2	−0,19	−1,39	−3,43
F7	Luteolin-7-*O*-glucoside	3	0,29	11	7	190,28	1,46	−0,24	−2,1
F8	Apigenin-7-*O*-glucoside	2	0,29	10	6	170,05	1,81	0,05	−1,61
Subclass: Flavanone glycoside									
F9	Hesperidin	4	0,54	15	8	234,29	−0,14	−1,48	−3,04
F10	Homoplantaginin	1	0,32	11	6	179,28	0,83	0,06	−1,89
Subclass: Flavone glucuronide									
F11	Luteolin 7-*O*-glucuronide	6	0,24	12	7	207,35	0,97	−0,15	−2,12
F12	luteolin 3′-acetyl-*O*-glucuronide	4	0,24	12	7	207,35	1,22	−0,15	−2,12
Subclass: Flavonoid									
F13	Gallocatechin	2	0,2	7	6	130,61	0	0,93	−0,29
F14	Isorhamnetin	2	0,06	7	4	120,36	1,87	2,29	−0,31
Subclass: Flavonoid glycoside									
F15	Hesperidin-7-*O*-glucoside	2	0,41	11	6	175,37	0,8	−0,33	−1,7
Subclass: Flavonol (Flavonoid)									
F16	Quercetin	3	0	7	5	131,36	1,54	1,99	−0,56

**Class: Diterpenes**									
Subclass: Diterpene (Terpenoid)									

D1	Methyl carnostate	6	0,67	4	2	66,76	5,22	4,4	3,48
D2	Carnosic acid	6	0,65	4	3	77,76	4,89	4,32	3,25
D3	Carnosol	6	0,65	4	2	66,76	4,38	3,96	3,25
D4	Rosmanol	6	0,65	5	3	86,99	3,41	2,93	2,42
D5	Rosmadial	4	0,55	5	1	80,67	3,31	3,51	2,25
D6	Epirosmanol methyl ether	4	0,63	3	2	57,53	4,72	4,72	3,06
D7	Rosmanol quinone	3	0,65	5	1	80,67	2,13	2,13	1,8
D8	Rosmaridiphenol	2	0,65	3	2	57,53	5,87	4,79	3,29
D9	2,3,4,4a,10,10a-Hexahidro-5,6-dihydroxy-1,1-dimethyl-7-(1-methylethyl)-9(1H)-Phenantrenone	1	0,63	3	2	57,53	4,72	4,72	3,06
D10	Epirosmanol	2	0,63	5	3	86,99	3,08	2,37	2,2
D11	Rosmanol methyl ether	2							

**Class: Hydroxycinnamic acids**									
Subclass: Phenolic acid									

PA1	Caffeic acid	6	0	4	3	77,76	1,15	1,09	0,7
PA2	Rosmarinic acid	6	0,11	8	8	144,52	2,36	1,65	0,9
PA3	*p*-Coumaric acid	3	0	3	2	57,53	1,46	1,38	1,28
PA4	Salvianolic acid B	2	0,17	16	9	278,04	3,98	2,9	0,25
PA5	Neochlorogenic acid	3	0,38	9	6	164,75	−0,42	−0,75	−1,05

N	Compound	Pharmacokinetics					Druglikeness	Medicinal Chemistry
		GI	BBB	P-gp	Water Sol (Esol)	skin permeation	Bioavai. Score	Synthetic accessibility
**Class: Flavonoids**								
Subclass: Flavone (Flavonoid)								

F1	Luteolin	H	No	No	−3,71	−6.25	0,55	3,02
F2	Apigenin	H	No	No	−3,94	−5.80	0,55	2,96
F3	Pectolinarigenin	H	No	No	−4,2	−5.86	0,55	3,22
F4	Cirsimaritin	H	No	No	−4,2	−5.86	0,55	3,27
F5	Genkwanin	H	No	No	−4,14	−5.66	0,55	3,03
Subclass: Flavone glycoside								
F6	Luteolin 7-*O*-rutinoside	L	No	Yes	−3,29	−10.06	0,17	6,36
F7	Luteolin-7-*O*-glucoside	L	No	Yes	−3,56	−8.00	0,17	5,17
F8	Apigenin-7-*O*-glucoside	L	No	Yes	−3,78	−7.65	0,55	5,12
Subclass: Flavanone glycoside								
F9	Hesperidin	L	No	Yes	−3,28	−10.12	0,17	6,34
F10	Homoplantaginin	L	No	Yes	−3,26	−8.53	0,17	5,31
Subclass: Flavone glucuronide								
F11	Luteolin 7-*O*-glucuronide	L	No	Yes	−3,41	−8.43	0,11	5,11
F12	luteolin 3′-acetyl-*O*-glucuronide	L	No	Yes	−3,57	−8.25	0,11	5,04
Subclass: Flavonoid								
F13	Gallocatechin	H	No	No	−2,08	−8.17	0,55	3,53
F14	Isorhamnetin	H	No	No	−3,36	−6.90	0,55	3,26
Subclass: Flavonoid glycoside								
F15	Hesperidin-7-*O*-glucoside	L	No	Yes	−3,16	−8.56	0,17	5,17
Subclass: Flavonol (Flavonoid)								
F16	Quercetin	H	No	No	−3,16	−7.05	0,55	3,23

**Class: Diterpenes**								
Subclass: Diterpene (Terpenoid)								

D1	Methyl carnostate	H	Yes	No	−5,26	−4.71	0,55	4,05
D2	Carnosic acid	H	No	No	−5,03	−4.86	0,56	3,81
D3	Carnosol	H	yes	Yes	−4,77	−5.21	0,55	4,88
D4	Rosmanol	H	No	Yes	−4,25	−5.99	0,55	5,07
D5	Rosmadial	H	No	Yes	−4,04	−6.05	0,55	4,51
D6	Epirosmanol methyl ether	H	Yes	Yes	−4,82	−4.79	0,55	3,5
D7	Rosmanol quinone	H	No	Yes	−3,25	−6.89	0,55	5,71
D8	Rosmaridiphenol	H	Yes	No	−5,63	−4.06	0,55	3,59
D9	2,3,4,4a,10,10a-Hexahidro-5,6-dihydroxy-1,1-dimethyl-7-(1-methylethyl)-9(1H)-Phenantrenone	H	Yes	Yes	−4,82	−4.79	0,55	3,5
D10	Epirosmanol	H	No	Yes	−3,96	−5.99	0,55	4,96
D11	Rosmanol methyl ether							

**Class: Hydroxycinnamic acids**								
Subclass: Phenolic acid								

PA1	Caffeic acid	H	No	No	−1,89	−6.58	0,56	1,81
PA2	Rosmarinic acid	L	No	No	−3,44	−6.82	0,56	3,38
PA3	*p*-Coumaric acid	H	No	No	−2,02	−6.26	0,85	1,61
PA4	Salvianolic acid B	L	No	No	−6,22	−7.86	0,11	6
PA5	Neochlorogenic acid	L	No	No	−1,62	−8.76	0,11	4,16

H: High, L: low, MW: molecular weight; Csp3: Fraction Csp3; HBA: H-bond acceptors; HBD: H-bond donors; TPSA (Å2): topological Polar Surface Area; LOGP: n-octanol/water partition coefficient (log Po/w); ESOL: Estimated Solubility; GI: Gastrointestinal Absorption; BBB: Blood–Brain Barrier; Pgp: P-glycoprotein substrates; Bioav. Score: Bioavailability Score; Synt. Acc.: Synthetic Accessibility; Water Sol. ESOL Log S; Drugliken. Bioac score; Medic Chem Synt. Acc.

The ADME predictions show high gastrointestinal (GI) absorption for flavonone (flavonoids) and low GI for flavonone (glycosides). Flavonone (flavonoids) (e.g., luteoline, found in three extracts) often exhibit higher bioavailability, whereas flavonone (glycosides) (e.g., luteoline-7-*o*-tutinoside, found in all extracts) generally show lower bioavailability [27].

**Fig. 3:**
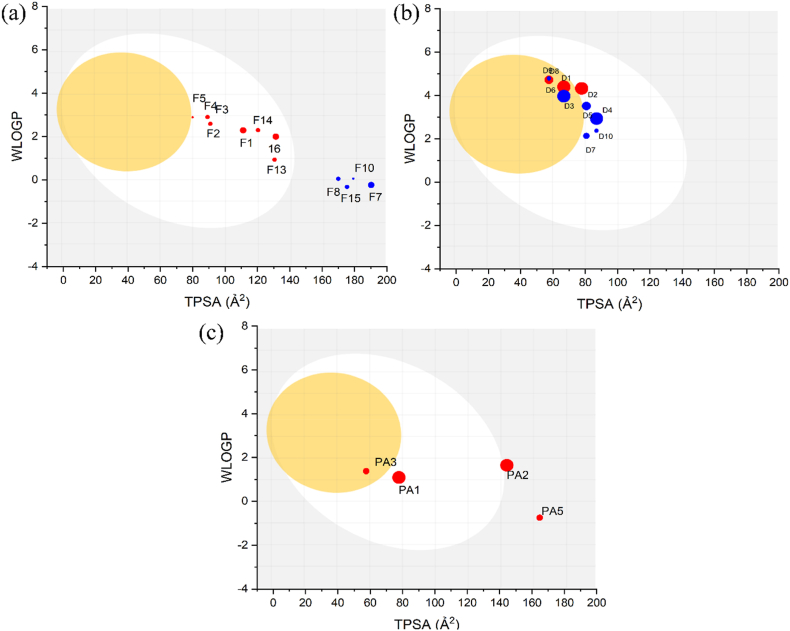
Boiled-Egg scheme of the identified components in *Rosmarinic officinalis L.* (a): Flavonoids Class, (b): Diterpenes calss, (c): Phenolic acid class. (The white region is for a high probability of passive absorption by GI and the yellow area (yolk) is for a high probability of brain penetration. Points colored in blue are predicted as actively effluxes by P-gp (PGP+) and in red if predicted as non-substrate of P-gp (PGP-); The size of the points is relative to the detection score of components in the six extracts).

The Boiled-Egg model (Fig. 3) was used to evaluate the gastrointestinal (GI) absorption and blood-brain barrier (BBB) permeability of all identified compounds. The results indicate that the compounds within each class demonstrate variable intestinal absorption.

Of the 16 flavonoids identified, only eight are non-substrates of P-gp, and are located in the white zone (Fig. 3a). These metabolites may be suitable for intestinal absorption, however their scores are relatively low and their distribution across extracts is heterogeneous. Fig. 3b shows that 5 diterpenes are located in the yellow zone, indicating their ability to cross the blood-brain barrier. Two of these diterpenes are P-gp substrates, and two are detected in all extracts, including one P-gp substrate. In the white zone are five diterpenes were identified, including one P-gp and one P + gp substrate with a high score. For phenolic acids, five compounds were detected, two are in the yellow zone, including one with a high score, while two are located in the white zone (Fig. 3c), including one with a high score. All four componds are predicted to be P-gp. Substrates.

**Fig. 4:**
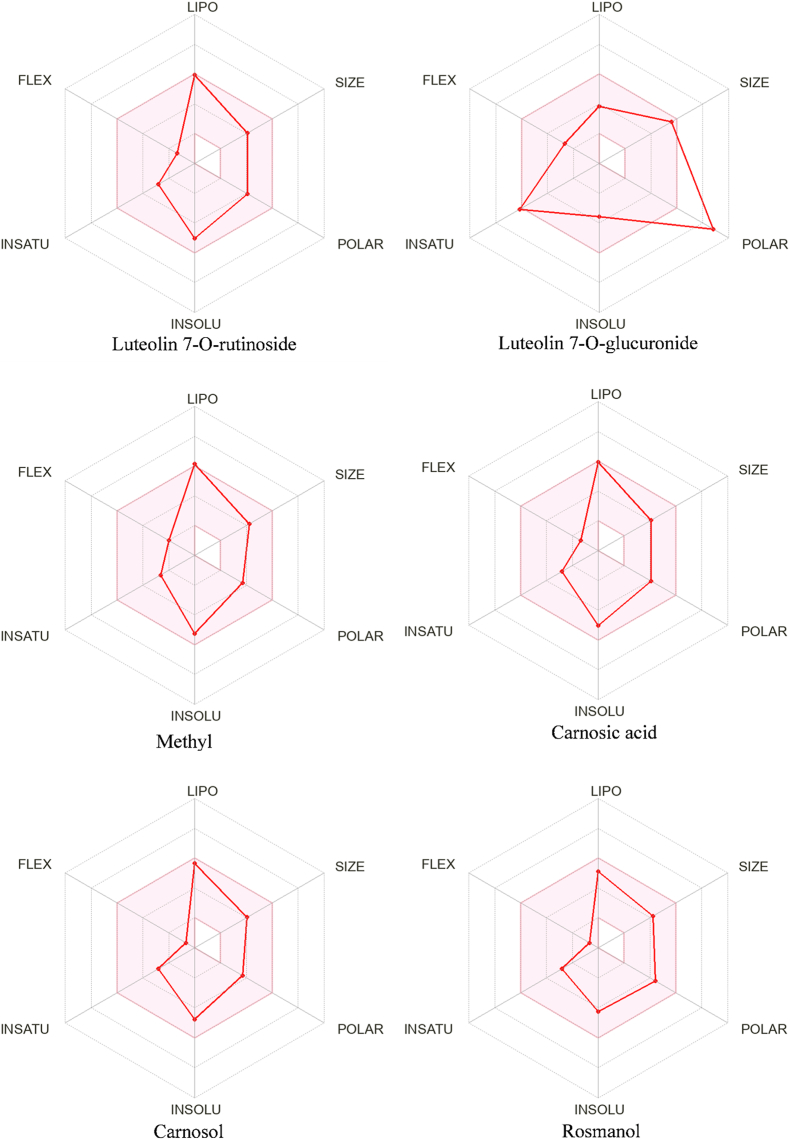
The bioavailability radar of the components of score raised in rosemary extracts.

The bioavailability radar of metabolites detected in extracts (score = 6) is given in Fig. 4. The optimal range is represented by the pink zone, which defines the ideal physicochemical domain for drug-like molecules [82]. Only the Luteolin 7-*O*-Glucuronide compound does not meet the criteria of oral bioavailability.

#### 3.7.1 Correlation between antioxidant, antimicrobial properties and ADME activity

Rosmarinic Acid, a well-established contributor to the antioxidant activity of rosemary [83], was detected in all extracts. However, its predicted ADME properties (Table 5) reveal certain limitations. Despite being classified as having “High” GI absorption, its WlogP value and predicted water solubility suggest that passive absorption may be restricted. Furthermore, rosmarinic acid is subject to extensive first-pass metabolism [84], which may significantly reduce its systemic bioavailability.

Similarly, carnosic acid and carnosol, both identified in all extracts, are known for their potent antioxidant [10]. Compared to rosmarinic acid, their predicted ADME profiles are more favorable, particularly due to higher lipophilicity (WLogP values), which generally enhances membrane permeability and absorption. However, both compounds are predicted to lack blood-brain barrier (BBB) permeability.

In the UAE leaf extract, elevated levels of carnosic acid, carnosol, and luteolin were observed. Their WLogP values, ranging from 3.25 to 4.89, support efficient interaction with lipid membranes, facilitating radical scavenging activity. While their low aqueous solubility may limit systemic bioavailability, this characteristic enhances their effectiveness *in vitro*, particularly in lipid-rich biological environments.

Extracts obtained by maceration (ME), from both leaves and stems, exhibited the lowest FRAP values, which correlate with their comparatively weaker predicted ADME properties. A similar trend is observed with ABTS assay results, suggesting a link between chemical bioavailability and antioxidant performance. Flavonoids, known for their safety and broad-spectrum antibacterial activity, play a significant role against both Gram-positive and Gram-negative bacteria [85]. Carnosic acid, carnosol, and structurally related diterpenes are likely key contributors to the observed antimicrobial effects. Their predicted high lipophilicity (WLogP) may enhance membrane permeability and absorption; however, their susceptibility to metabolic transformation and elimination warrants further investigation.

Methylation of these compounds reduces polarity, increases WLogP, and improves metabolic stability reflected in a bioavailability score of 0.55. This modification likely contributes to their persistence and strong performance in antioxidant assays, particularly in SE-derived extracts.

The presence of glycosides in the ME stems extracts, characterized by high water solubility and low gastrointestinal absorption, may explain their *in vitro* efficacy. The early inhibition against *E. coli* Gram-negative (G-) [86] by UAE and ME stems extracts is likely associated with the presence of carnosol and rosmarinic acid. Their moderate lipophilicity facilitates permeation through the outer membrane of *E. coli*. R, while rosmarinic acid's high topological polar surface area (TPSA) enables interaction with porins, promoting cellular entry. Maximum inhibition against *S. scabies* Gram positive (G+) observed with UAE leaf extracts can be attributed to the presence of epirosmanol methyl ether and rosmaridiphenol. These compounds exhibit high lipophilicity, which enhances their accumulation within the thick peptidoglycan layer of Gram-positive bacteria, ultimately leading to membrane disruption [87,88].

The impact of the extraction methods on compound composition, ADME properties, antioxidant activity, and antimicrobial effects is summarized in Table S2 in Supplementary Data. The synergistic and antagonistic interactions among the bioactive phytochemicals identified in rosemary extracts may modulate their antioxidant efficacy and free radical scavenging capacity. Synergies interactions between diterpenes and polyphenols enhance both radical-scavenging and membrane-disrupting activities.

Notably, the combination of carnosol and rosmarinic acid may have synergistic effects, as suggested by Farhadi et al. [89] by targeting bacterial proteins. carnosol (score = 6), with high solubility, enhances membrane penetration, whereas rosmarinic acid (score = 6), with a high TPSA = 144.52 Å^2^ (Table 5), facilitates interaction with bacterial targets. However, the co-presence of flavonoids glycosides (low GI absorption) and diterpenes (Terpenoid) (high GI absorption) may delay diterpene absorption, thereby reducing antimicrobial efficacy at lower doses.

### 3.8 Multivariate analysis

Multivariate analysis (HCA, PCA, MCA) was used to segment the six extraction methods {LME, LUAE, LSE, SME, SUAE, SSE} into distinct, homogeneous groups based on the 32 metabolites identified.

#### 3.8.1 Principal Component Analysis (PCA)

**Fig. 5:**
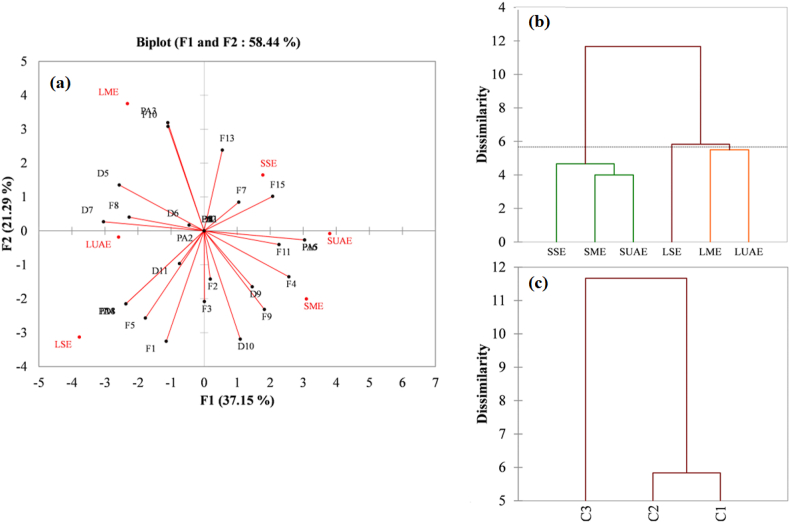
(a) Factorial maps: F1–F2 biplot, (b) hierarchical Clustering dendrogram using Ward's extracted method, (c) Cluster. F: Flavonoid; D: Diterpenes; PA: Phenolic acid; LME: leaf maceration extract; LUAE: leaf ultrasonic assisted extract; LSE: leaf soxhlet extract; SME: stem maceration extract; SUAE: stem ultrasonic assisted extract; SSE: stem soxhlet extract.

PCA is performed on the correlation matrix (Pearson), which standardizes variables. The F1–F2 biplot (Fig. 5a) displays observations and variables. F1 and F2 explain 58.44% of total variance. F1 positively correlates with nine flavonoids, two diterpenes, and one phenolic acid. Strong correlations are with PA5, F16, F4, F11, and F15. Conversely, F1 negatively correlates with five flavonoids, five diterpenes, and one phenolic acid, with strong negative correlations observed for D7, D5, D8, F14, and PA4. F1 contrasts {SME, SUAE, SSE} (positive scores) with {LME, LUAE, LSE} (negative scores). F2 positively correlates with five flavonoids, three diterpenes, and one phenolic acid, with strong positive correlations observed for PA3, F10, F13, D5, and F15. Conservly F2 negatively correlates with nine flavonoids, four diterpenes, and two phenolic acids, with strong negative correlations observed for F1, D10, F5, F9, D8, F14, and PA4. F2 separates LME (high positive score) from LSE (high negative score).

The biplot shows plant part-driven separation along F1. Leaf extracts cluster negatively, whereas stem extracts positively [90,91]. This axis highlights organ-specific chemistry in *Rosmarinus officinalis*. Stems accumulate lipophilic flavonoids and diterpenes such as carnosic acid and carnosol. Whereas leaves are enriched in phenolic acids such as rosmarinic and caffeic acid derivatives [63,92]. Loadings confirm stem samples correlate with diterpene markers.

F2 captures extraction method variability within leaf samples. It differentiates conventional and modern techniques. Soxhlet extraction (LSE) recovers less-polar compounds via prolonged heating [93]. Gentler methods such as maceration (LME) and ultrasound-assisted extraction (LUAE) preferentially extract polar metabolites. Positive F2 correlations for PA3 and F10 align with LME, whereas negative loadings for F1 and D10 associate with LSE [94,95].

These patterns arise from metabolite solubility and extraction physics. Polarity gradients between parts interact with method selectivity. High-temperature LSE enhances non-polar, thermostable compounds, whereas maceration and LUAE are better suited for polar, thermolabile molecules [93,95]. Selectivity depends on tissue biochemistry and extrinsic parameters like temperature, solvent polarity, and energy input.

ADME properties in Table 5, like LogP and TPSA, provide a molecular rationale. Lipophilic diterpenes with high LogP co-extract with stems and Soxhlet extraction, whereas hydrophilic glycosides with high TPSA are associated with leaves and gentler methods. Microwave-assisted methods further demonstrate that energy input and temperature influence metabolite profiles [58,96]. Additional modulating factors include environmental conditions and plant maturity [97]. Chemoprofiles therefore reflect interactions among genetics, physiology, and processing conditions. This understanding contributes to standardizing protocols for consistent, application-specific extracts.

#### 3.8.2 Hierarchical Cluster Analysis (HCA)

A Hierarchical Cluster Analysis was performed using Ward's method and Euclidean distance. This approach aims to minimize within-cluster variance at each step, forming compact and relatively homogeneous clusters. The dendrogram structure (Fig. 5b and c) revealed three main clusters (C1, C2 and C3):

LME and LUAE formed a closely related group (cluster C1), suggesting a high level of similarity in their metabolite composition. Extracts obtained by both methods contained two identical flavonoids, six diterpenes and three phenolic acids.

LSE stood alone, indicating a significantly distinct profile with the other extracts (cluster C2). This method produced a unique metabolite profile, likely reflecting the influence of prolonged heating and solvent recirculation associated with Soxhlet extraction.

SME, SUAE, and SSE grouped together, showing strong internal cohesion (cluster C3). Extracts obtained using these three methods contain four identical flavonoids, four diterpenes and three phenolic acids, further supporting their chemical similarity.

The intra-class variance (4.722; 74.56%) was substantially higher than the inter-class variance (1.611; 25.44%) (Table 6). A high intra-class variance indicates strong homogeneity within clusters, whereas inter-class variance reflect a degree of separation between clusters. These results reinforce the stability and reliability of the resulting classification.

**Table 6:** Variance decomposition for optimal classification.

	Absolu	Percentage (%)
Intra-class	4.722	74.56
Inter-class	1.611	25.44
Total	6.333	100

#### 3.8.3 Multiple Correspondence Analysis (MCA)

**Fig. 6:**
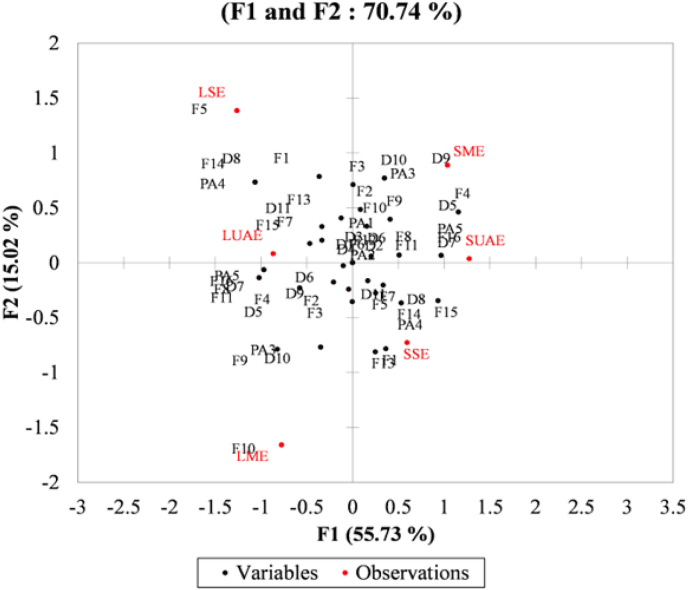
Multiple Correspondence Analysis (MCA) factorial plane (F1–F2) illustrating the clustering of observations and contribution of variables. F: Flavonoid; D: Diterpenes; PA: Phenolic acid; LME: leaf maceration extract; LUAE: leaf ultrasonic assisted extract; LSE: leaf soxhlet extract; SME: stem maceration extract; SUAE: stem ultrasonic assisted extract; SSE: stem soxhlet extract.

To visualize the multidimensional relationships among the variables and observations, an MCA was conducted (Fig. 6). The total inertia observed was low (0.75). The first two factor axes (F1 and F2) explain 58.44% of the total inertia and 70.74% of the adjusted inertia, which provides a good two-dimensional representation for this type of data. The first two dimensions of the MCA capture a significant portion of the structural variability, with specific variables such as F16 demonstrating an exceptionally high representation (cos^2^ > 0.93) on the primary axis F1. The most discriminating variables on F1 (55.7% adjusted inertia) are F5, F4 and D5 whereas F10, F5, D9 and F13 strongly influence F2. Furthermore, contribution analysis reveals that variables D9 and F13 are among the most influential in defining the F2 secondary axis, underscoring their important role in subgroup differentiation within leaf-based extracts.

The symmetrical and asymmetrical plots show the factorial map (F1–F2). these plots confirm the proximity of LME and LUAE (F1 < 0 and F2 > 0), in agreement with the HCA results and highlight the associations between variable categories. LSE appears isolated (F1 < 0 and F2 < 0), reinforcing its unique position in the dataset. Conversely, SME, SUAE, and SSE remained close in the factorial space, further supporting the existence of a cohesive group (F1 > 0).

The analyses (HCA, PCA, MCA) converge toward the existence of well-differentiated clusters among observations. There is a clear distinction between two main groups depending on the part of the rosemary analyzed: G1 = {LME, LUAE, LSE} and G2 = {SME, SUAE, SSE}. The main axis of differentiation (F1 in PCA, F1 in MCA) separates these two groups according to the types of metabolites identified. SME, SUAE, and SSE formed a homogeneous group, likely due to shared categorical characteristics. The second axis (F2 in PCA, F2 in MCA) introduces a distinction within G1, separating LSE from LME-LUAE. LME and LUAE consistently clustered together, sharing a common chemical and structural profile, whereas LSE stands out as a unique and potentially atypical profile.

#### 3.8.4 Extraction method and Plant-Part Effects

ANOVA was used to evaluate the effect of extraction method and plant-part on yield and various antioxidant assays. Post-hoc Tukey HSD Tests were applied to identify specific differences. Results indicated that antioxidant responses depend on both the extraction protocol and the plant part analyzed. This assay- and matrix-dependence is commonly reported for FRAP/ABTS-type measurements. Similar dependencies are documented for comparisons among extraction methods in leaf [98].

The extraction method significantly affected extraction yield. SE produced the lowest mean yield at 9.57. UAE yielded 15.7. ME achieved the highest yield at 17.2. Plant type also significantly influenced yield. Leaves yielded 15.9, exceeding stems by 4.00 units. Stems yielded only 11.9. Tukey HSD identified specific method x plant-part combinations driving these differences. These findings align with frequent reports demonstrating that extraction technique markedly influences compound recovery and apparent yield [99].

The extraction method was the dominant factor for FRAP (p < 0.0001). The Method × Type interaction was significant (p = 0.006). ME produced the lowest mean FRAP value at 0.734. UAE yielded 1.367. SE achieved the highest mean at 1.423. The lowest mean cell value occurred with ME x Leaves at 0.495. The highest values were observed for UAE x Leaves at 1.498 and SE x Leaves at 1.447. Tukey HSD results showed ME differed significantly from both UAE and SE (p < 0.0001).

For ABTS assay results, the extraction method, plant type, and their interaction were all highly significant for ABTS (all p < 0.0001). ME produced a mean of 0.050, which was substantially higher than UAE and SE. UAE yielded only 0.007. SE produced 0.005. Leaves showed a mean value of 0.033, exceeding stems at 0.008. The dominant condition was ME x Leaves at 0.089. Numerous Tukey contrasts were significant at p < 0.0001. These results are consistent with frequent reports of higher antioxidant potential in leaves [100].

For TCP Analysis, the extraction method showed strong significance (p < 0.0001). Plant type was also significant (p = 0.037), and the method × Type interaction was highly significant (p < 0.0001). The overall ranking by method was SE > UAE > ME. SE produced 0.939. UAE yielded 0.674. ME achieved 0.511. Stems showed a mean value of 0.739, slightly exceeding leaves at 0.677. Tukey HSD revealed significant method differences, typically at p ≤ 0.0001. The model fit was excellent with R^2^ = 0.964.

For TFC Results, plant type was the primary driver for TFC (p < 0.0001). The extraction method showed a smaller but significant effect (p = 0.002). The interaction was not significant (p = 0.278). Leaves contained more flavonoids at 0.512 compared to stems at 0.411. Tukey HSD highlighted differences between Leaves versus Stems and ME vs SE. This pattern matches common reported plant-part distributions for flavonoid richness [101].

For DPPH Radical Scavenging Activity, Method exerted a strong effect on DPPH (IC_50_ values). UAE showed the lowest IC_50_ at 14.1, indicating highest potency. SE produced an intermediate IC_50_ of 21.8. Whereas ME yielded the highest IC_50_ at 53.9, indicating lowest potency. Leaves showed a mean IC_50_ of 32.1, while stems exhibited a mean IC_50_ of 27.8. The Tukey ranking from lowest to highest was: SE*Leaves (9.91) < UAE*Stems (11.9) < UAE*Leaves (16.3) < SE*Stems (33.7) < ME*Stems (37.7) ≪ ME*Leaves (70.0). These results are consistent with reported sensitivity of DPPH outcomes to extraction modality [102]. Leaves showed lower radical-scavenging potency by this metric, which contrasts with their higher antioxidant capacity observed in other assays.

The extraction method significantly affected all measured parameters including FRAP, ABTS, TCP, TFC, DPPH, and yield. Plant type showed significant effects across most assays. Method × Type interactions were significant for FRAP, ABTS, TCP, and DPPH, wheras the interaction was not significant for TFC. Different assays identified different optimal extraction methods and plant parts. The results demonstrate a common assay and matrix dependency for antioxidant measurements (Table 7). They confirmed that the extraction protocol and plant tissue selection have a decisive influence on antioxidant evaluation outcomes.

**Table 7:** Analysis of variance (ANOVA) for the effects of extraction method, sample type, and their interaction on yield and antioxidant responses.

Parameters		F-value	p-value	Significant	R^2^ (Model)	RMSE
Yield	Method	22.3	<0.0001	Yes	0.847	2.017
	Type	17.7	0.001	Yes		
	Method*Type	2.13	0.161	No		
FRAP	Method	33.1	<0.0001	Yes	0.874	0.163
	Type	0.001	0.483	No		
	Method*Type	8.182	0.006	Yes		
ABTS	Method	1121.9	<0.0001	Yes	0.998	0.002
	Type	780.6	<0.0001	Yes		
	Method*Type	925.7	<0.0001	Yes		
TCP	Method	88.3	<0.0001	Yes	0.964	0.056
	Type	5.47	0.037	Yes		
	Method*Type	70.1	<0.0001	Yes		
TFC	Method	116	0.002	Yes	0.903	0.023
	Type	85.8	<0.0001	Yes		
	Method*Type	1.43	0.278	No		
IC_50_	Method	7369459.3	<0.0001	Yes	1	0.016
	Type	229183.4	<0.0001	Yes		
	Method*Type	3268546.1	<0.0001	Yes		

## 4 Conclusion

This study provides a comprehensive comparison of maceration (ME), ultrasound-assisted extraction (UAE), and Soxhlet extraction (SE) for obtaining bioactive compounds from *Rosmarinus officinalis* L. leaves and stems. Results demonstrated that both the extraction method and plant fraction strongly influence phytochemical profiles, bioactivity, and predicted pharmacokinetics. This novelty lies in its integrated comparative evaluation of leaves versus stems, employing three distinct extraction methods (ME, UAE, and SE), and assessing both antioxidant and antimicrobial activities, further enhanced by the combination of ADME prediction, ANOVA, and multivariate statistical analysis.

UAE proved to be the most effective method for releasing antioxidant and antimicrobial diterpenes (e.g., carnosic acid) and aglycone flavonoids, showing the highest radical-scavenging and bacterial inhibition activities. SE yielded the greatest phenolic content but may promote degradation of thermolabile compounds, whereas ME favored polar glycosides with limited GI absorption yet potential for soluble formulations.

Multivariate analyses confirmed distinct chemometric clustering of extracts according to extraction technique and plant part, while *in silico* ADME predictions suggest that lipophilic diterpenes, enriched in UAE and SE extracts, may face solubility challenges for systemic delivery but could be promising candidates for topical formulations, pending experimental validation. Conversely, ME extracts, richer in hydrophilic glycosides, present a distinct pharmacokinetic profile that might require encapsulation strategies for oral bioavailability. Future studies should focus on validating the *in silico* ADME predictions through *in vivo* models, investigating the synergistic interactions between compounds (e.g., carnosic acid and rosmarinic acid) using combination assays, and developing optimized food-grade formulations (e.g., emulsions, edible coatings) to test the efficacy of selected extracts in real food matrices for shelf-life extension.

## Ethical statement - studies in humans and animals

Not applicable.

## CRediT authorship contribution statement

**Fatima-Zahrae Ed-darraz:** Conceptualization, Formal analysis, Investigation, Methodology, Resources, Writing – original draft. **Saida Tayibi:** Conceptualization, Formal analysis, Funding acquisition, Methodology, Resources, Supervision, Validation, Visualization, Writing – review & editing. **Sana Mounaimi:** Conceptualization, Funding acquisition, Investigation. **Asmae Hbika:** Conceptualization, Data curation, Methodology, Writing – original draft. **Meryem Boufetacha:** Data curation, Methodology, Resources, Validation. **Karim Lyamlouli:** Data curation, Formal analysis, Investigation, Methodology, Resources, Writing – review & editing. **Abdellatif Barakat:** Funding acquisition, Methodology, Resources, Validation, Writing – review & editing. **El Khadir Gharibi:** Conceptualization, Project administration, Supervision, Validation.

## Declaration of competing interest

The authors declare that they have no known competing financial interests or personal relationships that could have appeared to influence the work reported in this paper.

## Data Availability

No data was used for the research described in the article.
