# Supplementary material for: Multivariate analysis and ADME profiling of Rosmarinus officinalis L. leaf vs. stem extracts: Extraction method-driven bioactivity and pharmacokinetic implications
Source: Biochem Biophys Rep. 2026 Apr 16;46:102589. doi: 10.1016/j.bbrep.2026.102589 (PMC13101620; doi:10.1016/j.bbrep.2026.102589)
Supplement: Multimedia component 1 [file mmc1.docx]

**Supplementary material**

**Multivariate Analysis and ADME Profiling of *Rosmarinus officinalis* L. Leaf vs. Stem Extracts: Extraction Method-Driven Bioactivity and Pharmacokinetic Implications**


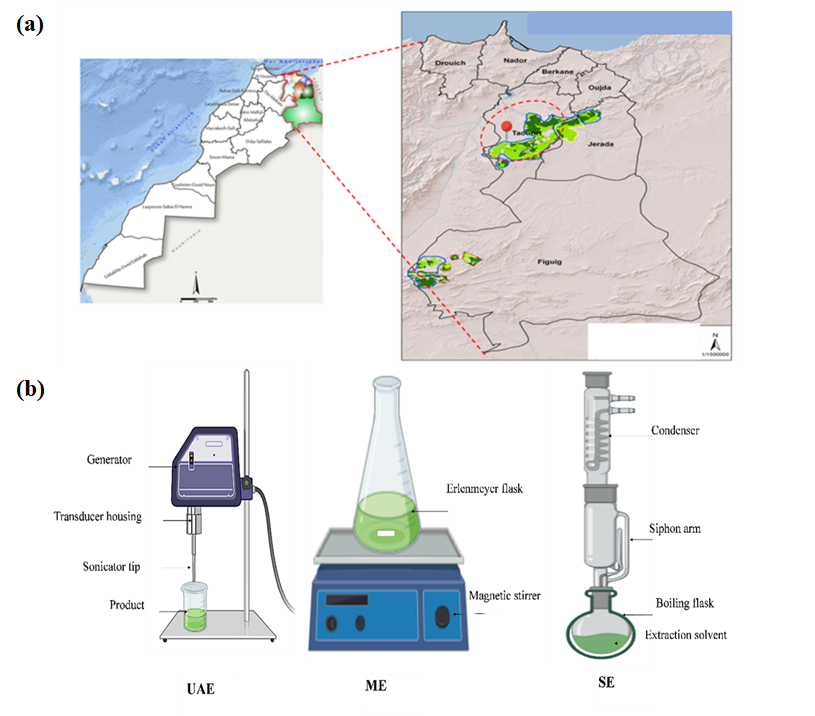


**Fig. S1.**  (a) Location of rormary's harvest area (Naggar and Iharchine, 2015), (b) Extraction methods using ultrasonic assisted (UAE), maceration (ME), and soxhlet (SE).

**
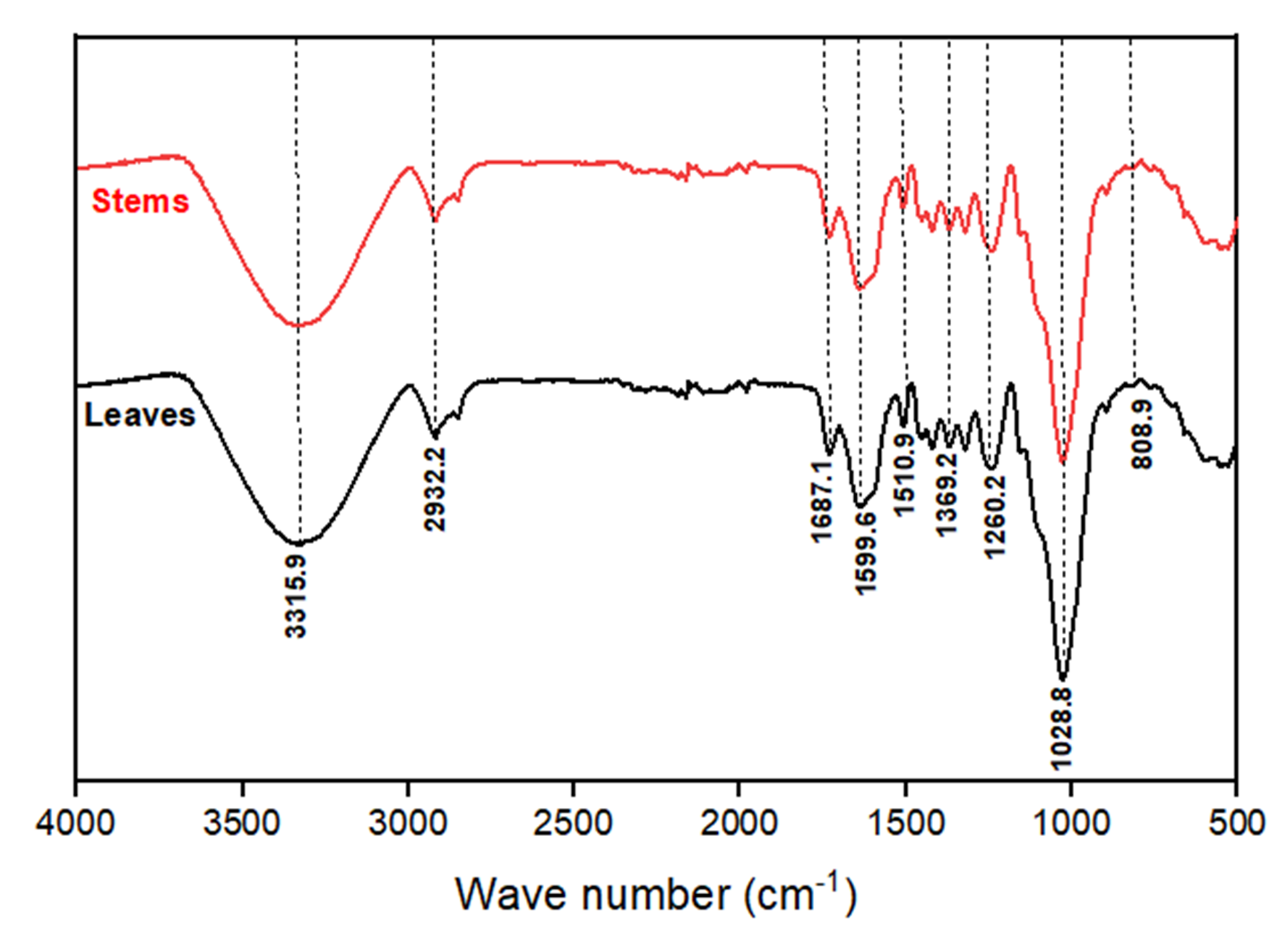
**

**Fig. S2.** ATR-FTIR spectra of rosemary leaves and stems before extraction


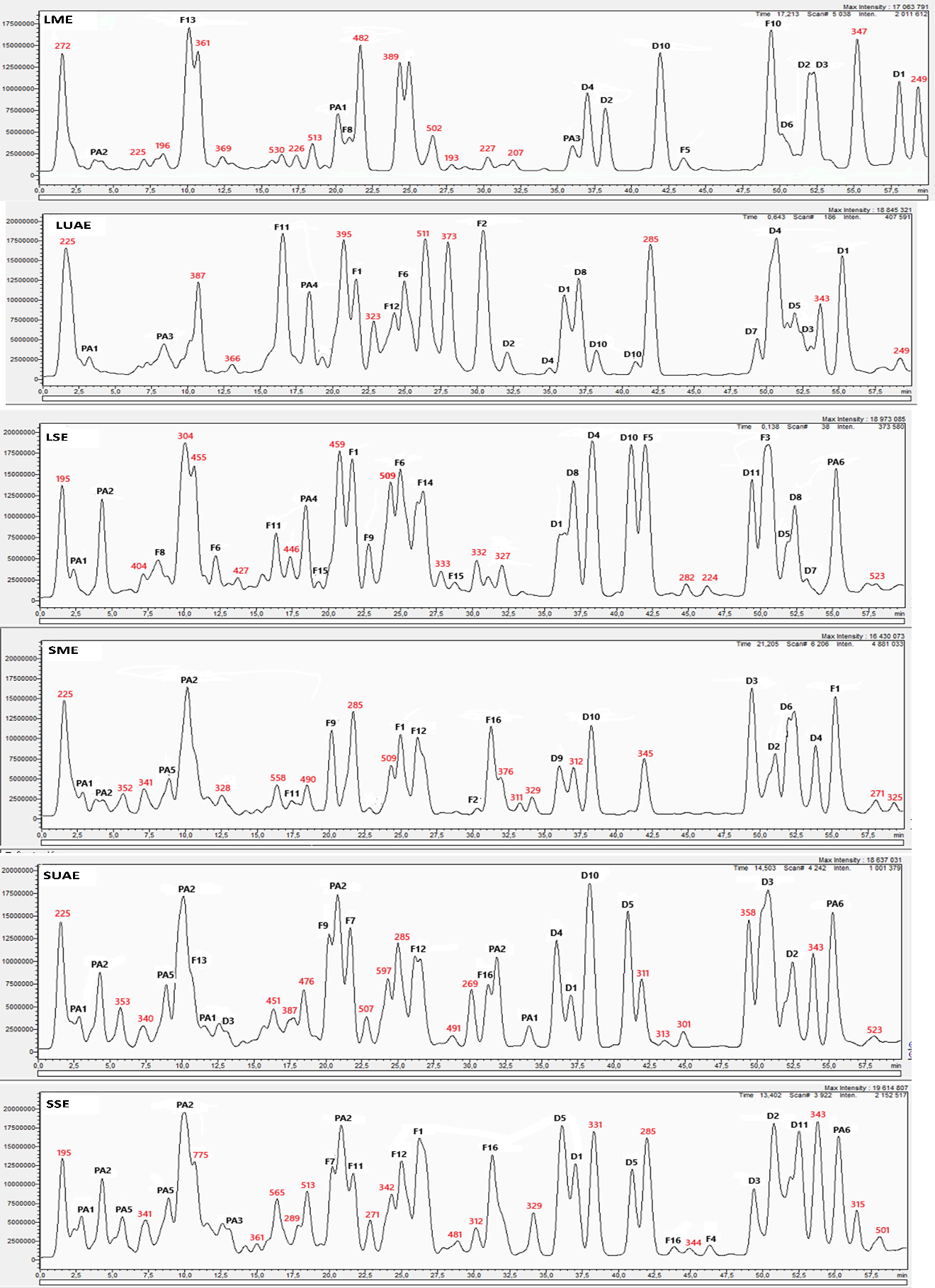


**Fig S3**. LC-MS spectra of rosemary leaves and stems extracts.


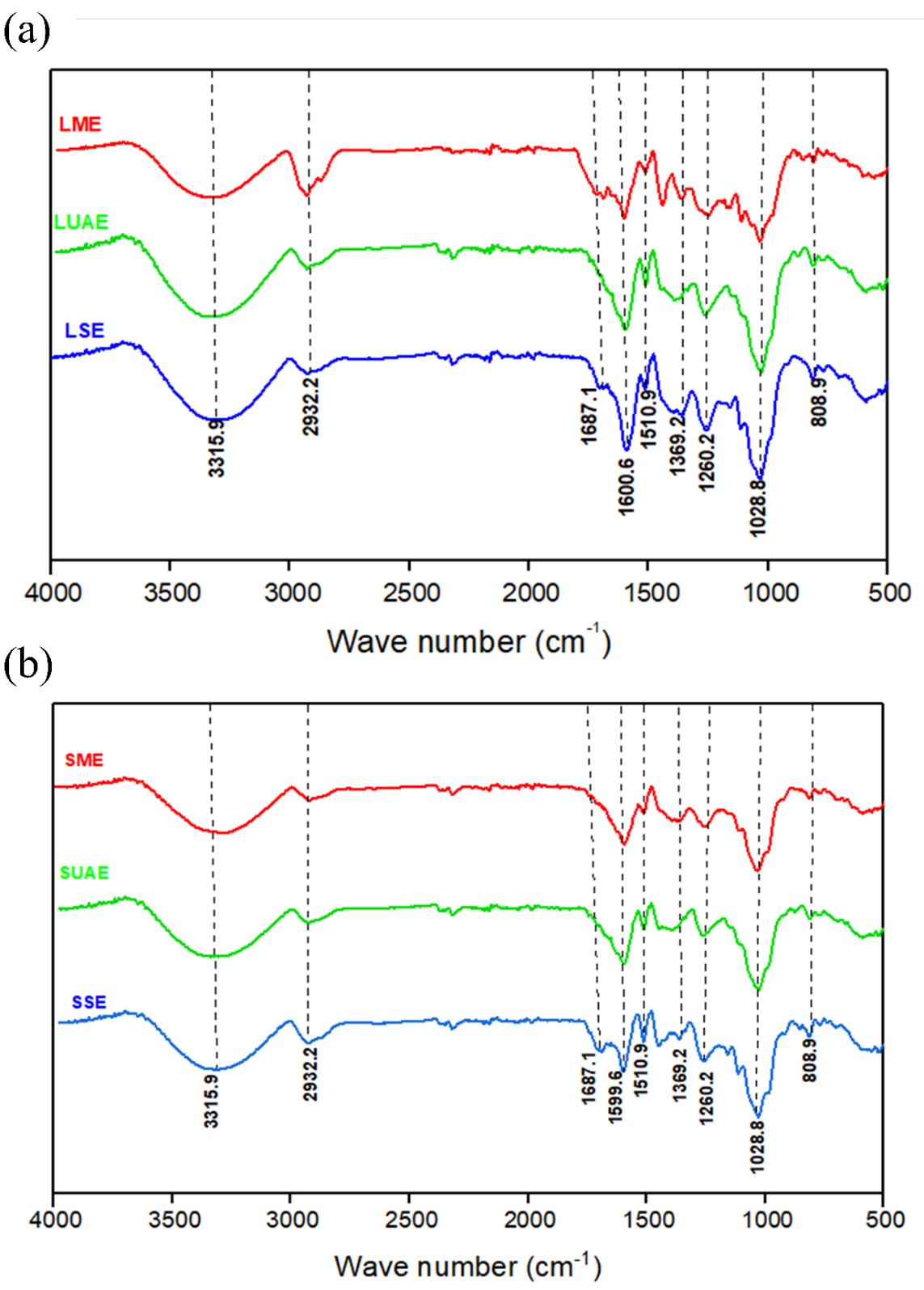


**Fig. S4.** ATR-FTIR spectra of rosemary (a) leaves and (b) stems extracts. LME: leaf maceration extract; LUAE: leaf ultrasonic assisted extract; LSE: leaf soxhlet extract; SME: stem maceration extract; SUAE: stem ultrasonic assisted extract; SSE: stem soxhlet extract.

**Table S1**

LC-MS identification of unidentified compounds in the extract of rosemary leaf and stem.

| [M-H]^-^ | m/z Main Fragments | LME | LUAE | LSE | SME | SUAE | SSE |
| --- | --- | --- | --- | --- | --- | --- | --- |
| 225 | 179-143-113 | X | X |  | X | X |  |
| 271 | 242-159-143 |  |  |  | X |  | X |
| 249 | 281-113 | X | X |  |  |  |  |
| 272 | 182-115 | X |  |  |  |  |  |
| 193 | 194-149 | X |  |  |  |  |  |
| 195 | 129 |  |  | X |  |  | X |
| 285 | 270-242-172-118 |  | X |  | X | X | X |
| 196 | 161 | X |  |  |  |  |  |
| 207 | 162-118 | X |  |  |  |  |  |
| 226 | 181-115 | X |  |  |  |  |  |
| 227 | 136 | X |  |  |  |  |  |
| 482 | 285-256-175-151 | X |  |  |  |  |  |
| 347 | 267-217 | X |  |  |  |  |  |
| 361 | 209-152 | X |  |  |  |  |  |
| 369 | 179-151 | X |  |  |  |  |  |
| 398 | 284-256-155-133 | X |  |  |  |  |  |
| 502 | 285 | X |  |  |  |  |  |
| 513 | 299-243-161 | X |  |  |  |  | X |
| 530 | 355-295-235-193-143 | X |  |  |  |  |  |
| 323 | 191-125 |  | X |  |  |  |  |
| 343 | 284-227-201 |  | X |  |  |  |  |
| 366 | 161-133 |  | X |  |  |  |  |
| 373 | 179-135 |  | X |  |  |  |  |
| 387 | 225-163 |  | X |  |  | X |  |
| 395 | 161-133 |  | X |  |  |  |  |
| 511 | 475 |  | X |  |  |  |  |
| 304 | 225-163-147 |  |  | X |  |  |  |
| 404 | 281-221-179-135 |  |  | X |  |  |  |
| 427 | 221-161-133 |  |  | X |  |  |  |
| 446 | 285 |  |  | X |  |  |  |
| 455 | 387-343-289-247-163 |  |  | X |  |  |  |
| 459 | 216-191-135 |  |  | X |  |  |  |
| 327 | 211-183-137 |  |  | X |  |  |  |
| 224 | 181 |  |  | X |  |  |  |
| 282 | 267-195-144 |  |  | X |  |  |  |
| 332 | 269-135 |  |  | X |  |  |  |
| 333 | 331-287-167-137 |  |  | X |  |  |  |
| 311 | 267-225-183-115 |  |  |  | X | X |  |
| 312 | 211-163-117 |  |  |  | X |  | X |
| 325 | 183-119 |  |  |  | X |  |  |
| 328 | 285-241-163-125 |  |  |  | X |  |  |
| 329 | 283-211-171-139 |  |  |  | X |  | X |
| 341 | 221-161-135 |  |  |  | X |  |  |
| 345 | 274-239 |  |  |  | X |  |  |
| 352 | 191-135 |  |  |  | X |  |  |
| 376 | 286-214-161-132 |  |  |  | X |  |  |
| 490 | 323-300-221-161 |  |  |  | X |  |  |
| 509 | 311-267 |  |  |  | X |  |  |
| 558 | 233-200-133 |  |  |  | X |  |  |
| 269 | 161 |  |  |  |  | X |  |
| 301 | 285-241-202 |  |  |  |  | X |  |
| 313 | 267-254-187 |  |  |  |  | X |  |
| 340 | 281-251-179 |  |  |  |  | X |  |
| 342 | 297-161 |  |  |  |  | X | X |
| 358 | 283-227 |  |  |  |  | X |  |
| 353 | 191-135 |  |  |  |  | X |  |
| 451 | 313-168-150-137 |  |  |  |  | X |  |
| 476 | 329-299-227-152 |  |  |  |  | X |  |
| 491 | 445-283 |  |  |  |  | X |  |
| 507 | 461-391-313 |  |  |  |  | X |  |
| 523 | 455 |  |  |  |  | X |  |
| 597 | 373-315 |  |  |  |  | X |  |
| 289 | 161-133 |  |  |  |  |  | X |
| 315 | 287 |  |  |  |  |  | X |
| 331 | 267-217-201 |  |  |  |  |  | X |
| 344 | 301 |  |  |  |  |  | X |
| 361 | 268-179-122 |  |  |  |  |  | X |
| 565 | 403-327-297-235-179-135 | |  |  |  |  | X |
| 341 | 221-161-135 |  |  |  |  |  | X |
| 481 | 268-240-117 |  |  |  |  |  | X |
| 501 | 455-334-287 |  |  |  |  |  | X |
| 775 | 347-163 |  |  |  |  |  | X |
| LME: leaf maceration extract; LUAE: leaf ultrasonic assisted extract; LSE: leaf soxhlet extract; SME: stem maceration extract; SUAE: stem ultrasonic assisted extract; SSE: stem soxhlet extract | | | | | | | |

**Table S2**

Impact of extraction methods on compound composition, ADME properties and bioactivity.

| **Method** | **Major Compounds** | **Key ADME Properties** | **Impact on Bioactivity** |
| --- | --- | --- | --- |
| ME | Glycosylated flavonoids,  Phenolic acids | High solubility  Low GI absorption | Moderate antioxidant activity,  Dose-dependent antimicrobial effects |
| UAE | Diterpenes,  Aglycone flavonoids | High lipophilicity  Metabolic stability | Superior antioxidant/antimicrobial activity |
| SE | Oxidized diterpenes,  Methylated flavonoids | Prolonged half-life  Reduced hepatic clearance | Persistent antioxidant activity,  Delayed antimicrobial effects |
| ME: maceration extraction, UAE: ultrasonic assisted extraction, SE: soxhlet extraction, GI: Gastrointestinal | | | |
